# Cytokimera GIL-11 rescued IL-6R deficient mice from partial hepatectomy-induced death by signaling via non-natural gp130:LIFR:IL-11R complexes

**DOI:** 10.1038/s42003-023-04768-4

**Published:** 2023-04-15

**Authors:** Puyan Rafii, Christiane Seibel, Hendrik T. Weitz, Julia Ettich, Anna Rita Minafra, Patrick Petzsch, Alexander Lang, Doreen M. Floss, Kristina Behnke, Karl Köhrer, Jens M. Moll, Jürgen Scheller

**Affiliations:** 1 Institute of Biochemistry and Molecular Biology II, Medical Faculty, Heinrich-Heine-University, 40225 Düsseldorf, Germany; 2 Biological and Medical Research Center (BMFZ), Medical Faculty, Heinrich-Heine-University, Universitätsstraße 1, 40225 Duesseldorf, Germany; 3 Cardiovascular Research Laboratory, Medical Faculty, University Hospital Düsseldorf, 40225 Düsseldorf, Germany

**Keywords:** Interleukins, Interleukins

## Abstract

All except one cytokine of the Interleukin (IL-)6 family share glycoprotein (gp) 130 as the common β receptor chain. Whereas Interleukin (IL-)11 signal via the non-signaling IL-11 receptor (IL-11R) and gp130 homodimers, leukemia inhibitory factor (LIF) recruits gp130:LIF receptor (LIFR) heterodimers. Using IL-11 as a framework, we exchange the gp130-binding site III of IL-11 with the LIFR binding site III of LIF. The resulting synthetic cytokimera GIL-11 efficiently recruits the non-natural receptor signaling complex consisting of gp130, IL-11R and LIFR resulting in signal transduction and proliferation of factor-depending Ba/F3 cells. Besides LIF and IL-11, GIL-11 does not activate receptor complexes consisting of gp130:LIFR or gp130:IL-11R, respectively. Human GIL-11 shows cross-reactivity to mouse and rescued *IL-6R*^*−/−*^ mice following partial hepatectomy, demonstrating gp130:IL-11R:LIFR signaling efficiently induced liver regeneration. With the development of the cytokimera GIL-11, we devise the functional assembly of the non-natural cytokine receptor complex of gp130:IL-11R:LIFR.

## Abstract (web summary)

A human Cytokimera GIL-11 is generated, with specific high affinity activation of the non-natural receptor gp130:LIFR:IL-11R complex and can rescue IL-6R deficient mice from death following partial hepatectomy.

## Introduction

Progress made in our understanding of cytokine receptor complex formation has enabled the implementation of various of approaches to assemble non-natural cytokine receptor complexes by designer cytokines. These include synthekines^1^, neoleukins^2^ and chimeric cytokines (cytokimeras)^3^ but also fully synthetic cytokine systems^4^. Synthekines are fusions of two dominant negative cytokine variants in which each variant binds only one receptor subunit^1^. Neoleukins recapitulate some aspects of the natural cytokines but have a completely unrelated overall design and amino acid sequence^2^. Cytokimeras are based on the framework of a natural cytokine with at least one receptor recognition site exchanged from a closely related cytokine^3^, a concept that has yet specifically been applied for the IL-6 type cytokines Interleukin (IL-)6 and ciliary neurotrophic factor (CNTF) in cytokine IC7^3^.

IL-6-type cytokines comprise IL-6, IL-11, IL-27, IL-30, IL-31, leukemia inhibitory factor (LIF), oncostatin M (OSM), CNTF, cardiotrophin-1 (CT-1), cardiotrophin-like cytokine (CLC) and neuropoeitin^5,6^. Apart from IL-31, all IL-6-type cytokines induce signal transduction via the common gp130 β-receptor^7,8^, which activates signaling cascades including the JAK/STAT, Ras/Map kinase and phosphatidylinositol 3-kinase pathways^8,9^. Whereas IL-6 and IL-11 signal via gp130 homodimers, the other cytokines signal via gp130 heterodimers. For instance, CNTF and LIF recruit gp130:LIF receptor (LIFR)^10–12^. Moreover, the IL-6-type cytokines IL-6, IL-11 and CNTF have to interact with specific α-receptors (IL-6R, IL-11R, CNTFR, respectively) to allow binding to β-receptors^10,11,13–15^.

In the landmark paper from 1999^3^, Kallen et al. suggested that the receptor recognition sites of cytokines have evolved as discontinuous modules which should principally be freely exchangeable between different cytokines. In general IL-6 type cytokines have in the case of LIF two or the case of IL-11 or CNTF three receptor binding sites, the site I for the α-receptor, site II and site III for the two β-receptors^10–12^. Through the transfer of the LIFR binding site III of CNTF^16^ to binding site III of IL-6 the first of its kind cytokimera IC7 was described more than 20 years ago^3^. The abbreviation IC7 originates from the seventh tested chimeric Interleukin/CNTF variant. The module swap created a mash-up cytokine with IL-6R dependent activity on cells expressing gp130, IL-6R, and LIFR, instead of being dependent on IL-6R and gp130 in case of IL-6 or on gp130, CNTFR and LIFR in case of CNTF^3^. IC7 was recently shown to improve glucose tolerance and hyperglycemia, thereby preventing weight gain and liver steatosis in mice^17^. Apart from IC7 also other cytokines of the IL-6 cytokine family showed protective effects against obesity and insulin, including IL-6^18^, IL-27^19^ and CNTF^20^. However, any therapeutic use of IL-6 is, however, out of the question because of its pronounced pro-inflammatory effects. The CNTF variant Axokine failed because of the early development of neutralizing CNTF antibodies^21^. IC7 combined the best of both worlds and showed no safety issues in non-human primates^17^.

Here, we developed the cytokimera GIL-11 which binds the non-natural receptor complex consisting of gp130:IL-11R:LIFR. We used IL-11 as a backbone for the transfer of site III from LIF, resulting in the cytokimera GIL-11. The highly active GIL-11 combines the activities of LIF^22,23^ and IL-11^24^ in one molecule. Of note, a protective contribution of IL-11 in liver diseases has recently been challenged^25,26^, making the therapeutic application of IL-11 undesirably. To demonstrate the therapeutic potential of GIL-11, we made use of *IL-6R*^*−/−*^ mice challenged in partial hepatectomy (PHX). *IL-6R*^*−/−*^ mice showed a high mortality rate of up to 80% versus 10% in wild-type mice following PHX^27^. Here, we showed that GIL-11 rescued mice from death following partial hepatectomy.

## Results

### Cytokimera GIL-11: generation of chimeric IL-11:LIF synthetic cytokine

**Fig. 1:**
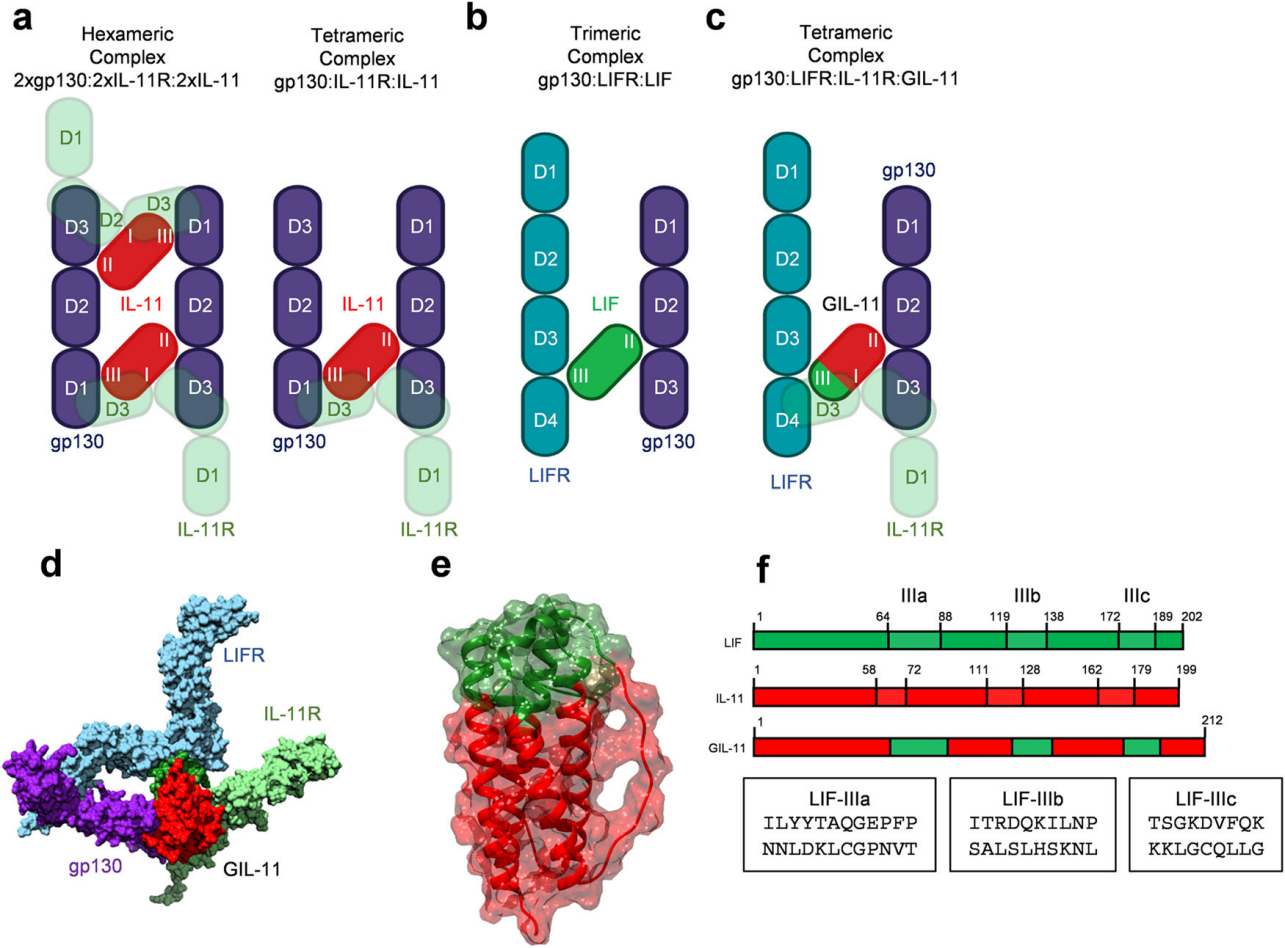
Design of the cytokimera GIL-11. **a** schematic illustration of the tetrameric and hexameric IL-11:IL-11R:gp130 receptor complex possibilities. IL-11 initially binds to IL-11R via site I. gp130 is recruited into this complex via major interactions between site III of IL-11 and D1 (Ig-like) of gp130 and site II of IL-11 and D2/D3 (cytokine binding module (CBM)) of gp130. **b** schematic illustration of the trimeric LIF:gp130:LIFR complex. LIF binds via site III to D3/D4 of LIFR and via site II to D2/D3 of gp130. **c** schematic illustration of the tetrameric GIL-11:IL-11R:gp130:LIFR complex. GIL-11 binds via site III to D3/D4 of LIFR, via site II to D2/D3 of gp130 and via site I to D2/D3 of IL-11R. **d** schematic illustration (left) and space fill model (right) of the tetrameric GIL-11:IL-11R:LIFR:gp130 (PDB 6O4P;^11^ 2Q7N;^83^ 1P9M^10^). GIL-11 binds via site I to IL-11R, via site II to gp130 and via site III to LIFR. **e** Combined transparent space fill/band model of GIL-11, red: IL-11, green: exchanged region by LIF. **f** Structure-based schematic sequence alignment of hIL-11 (PDB 6O4O^11^), hLIF (PDB 1PVH^33^) and the resulting GIL-11. Site IIIa,b,c residues are highlighted in green and red boxes.

The IL-6 type cytokines share a common four-helix bundle structure consisting of four anti-parallel alpha-helices (A, B, C, and D) connected by two long cross-over loops (AB, CD) and one short loop (BC)^28^. IL-6, IL-11 and CNTF bind to their α-receptor via binding site I which includes residues of the C-terminal AB loop and the C-terminal D-helix^10,11,13–15^. Residues of the A- and C-helices of CNTF, LIF, and IL-6 constitute a gp130-binding site which is called site II^10–12^. In IL-6 and IL-11 a second gp130-binding site III consists of amino acids residues of the N-terminal AB loop, the C-terminal CD loop, and the N-terminal D-helix^10,11^. Site III in CNTF and LIF renders the contact to LIFR, whereas site II is in contact with gp130^12^. For IL-11, site II and site III are in contact with gp130, whereas a third site (site I) recruits IL-11R^11^. Of Notably, primary binding of IL-11 to IL-11R is mandatory for secondary binding to gp130^11,29^. The site I,II,III paradigm for IL-11 and LIF is illustrated in Fig. 1a, b.

Of note IL-6 and IL-11 can form tetrameric and hexameric receptor complexes, consisting of one cytokine, one α-receptors and two gp130 or two cytokines, two α-receptors and two gp130^10,30^ (Fig. 1a). Albeit the tetrameric receptor complex is principally biologically active^31^, the structures of receptor complexes showed only hexameric assemblies^10,11^. However, LIF exclusively signals via a trimeric receptor complex consisting of LIF engaged with one gp130 via site II and one LIFR via site III^12,32^ (Fig. 1b). Of note, LIF does not require an α-receptor to bind to its β-receptor combination of gp130 and LIFR^12^. The binding of LIF to LIFR is, however, facilitated by an identically located binding site III in IL-11 to gp130 (Fig. 1b)^12^. We hypothesized that the structure-based exchange of the partitioned binding site III of human IL-11 with site III of human LIF will render the resulting chimeric cytokine into a binder of the non-natural cytokine receptor composition gp130:IL-11R:LIFR, a cytokine class which we called cytokimera GIL-11. Since β-receptor binding of LIF is α-receptor independent^12^, we were undecided if gp130:LIFR recruitment of the chimeric cytokine GIL-11 will be IL-11R dependent (Fig. 1c, d). Structural inspection of site III in IL-11 and LIF guided the design of the cytokimera GIL-11 with the framework of IL-11 and an exchange of site III from LIF^11,33^. IL-11 consists of 199 amino acids, we pinpointed the partitioned site III from amino acids 58–72 for IIIa, 111–128 for IIIb and 162–179 for IIIc. LIF has 202 amino acids with partitioned site III located from amino acids 64–88 for IIIa, 119–138 for IIIb and 172–189 for IIIc (Fig. 1d–f, Supplementary Fig. 1A). We decided to transfer the complete binding site from LIF to IL-11, albeit this resulted in somewhat longer GIL-11 cytokimera with 211 amino acids than the original IL-11. Molecular modeling suggested that the transfer of the entire site III amino acid stretches should not interfere with the overall architecture and folding of the cytokimera GIL-11 (Fig. 1d, e)^11,12^. Comparable to trimeric LIF:gp130:LIFR complexes, GIL-11 will only form tetrameric GIL-11:IL-11R:gp130:LIFR receptor complexes. The tetrameric assembly will be based on the requirement of GIL-11 for the LIFR. Whereas gp130 has two cytokine binding sites in one receptor molecule, LIFR has only one cytokine binding site. The LIFR interacts only with site III of LIF/GIL-11, whereas gp130 contacts site II of LIF/GIL-11. In this setting, the second contact site in gp130 remains free, because site III of LIF/GIL-11 cannot interact with gp130 (Fig. 1c) and the formation of hexameric 2xGIL-11:2xIL-11R:1xLIFR:1xgp130 complexes can be excluded.

### GIL-11 induce JAK/STAT signaling and cellular proliferation via the non-natural gp130:IL-11R:LIFR cytokine receptor complex

**Fig. 2:**
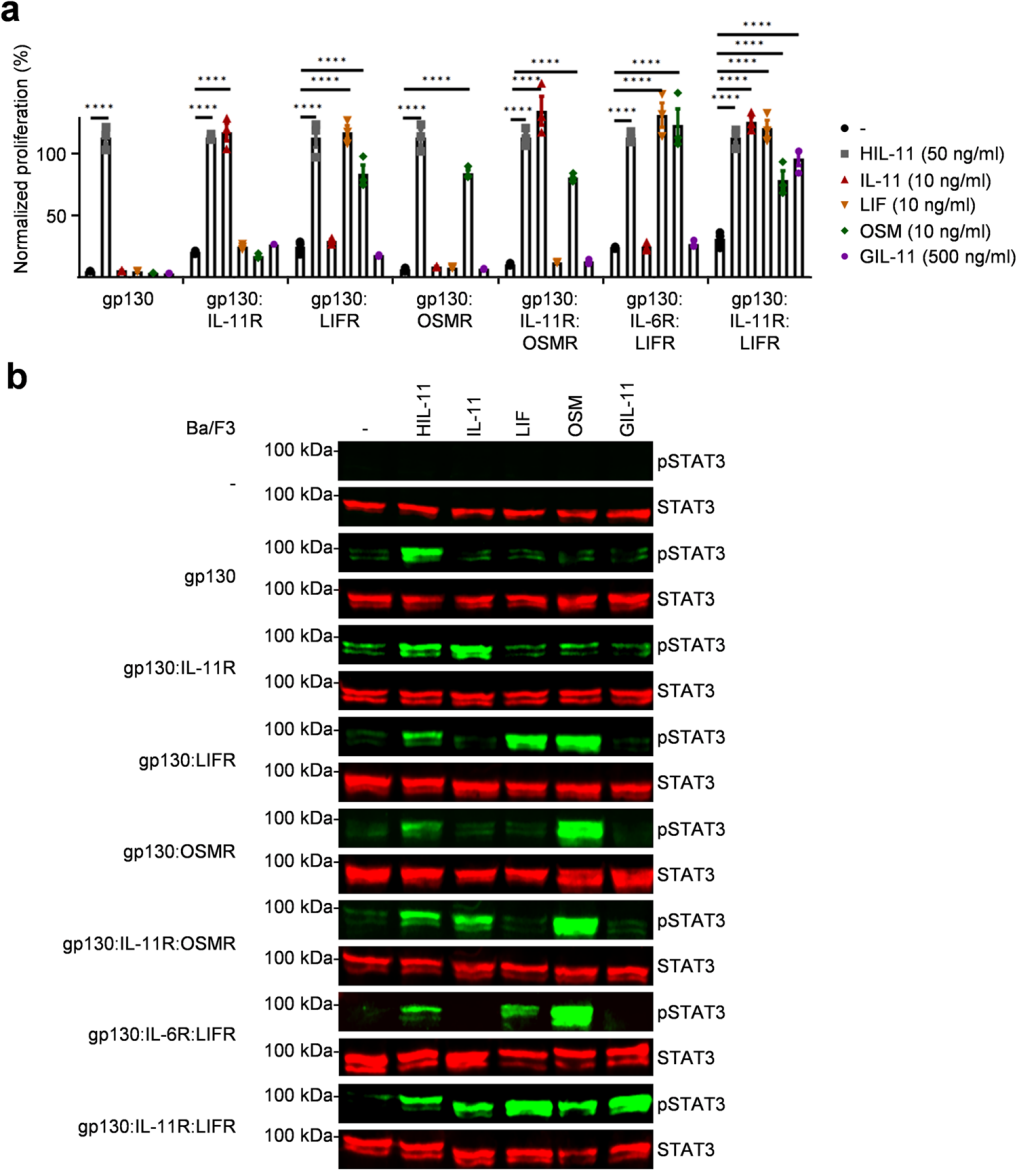
GIL-11 induce JAK/STAT signaling and cellular proliferation via the non-natural gp130:IL-11R:LIFR cytokine receptor complex. **a** Proliferation of Ba/F3-gp130, Ba/F3-gp130-IL-11R, Ba/F3-gp130:LIFR, Ba/F3-gp130:OSMR, Ba/F3-gp130:IL-11R:OSMR, Ba/F3-gp130:IL-6R:LIFR, Ba/F3-gp130:LIFR:IL-11R cells without cytokine (-), with 50 ng/ml HIL-11, 50 ng/ml IL-11, 50 ng/ml IL-6, 10 ng/ml LIF, 10 ng/ml OSM, 500 ng/ml GIL-11. Data represent ±SEM of three independent experiment out of three with three technical replicates each. Two-way ANOVA, including Dunnet’s correction was used for statistics. **b** STAT3 activation in Ba/F3, Ba/F3-gp130, Ba/F3-gp130_IL-11R, Ba/F3-gp130:LIFR, Ba/F3-gp130:OSMR, Ba/F3-gp130:IL-11R:OSMR, Ba/F3-gp130:IL-6R:LIFR, Ba/F3-gp130:LIFR:IL-11R cells without cytokine (-) and after stimulation with 50 ng/ml HIL-11, 50 ng/ml IL-11, 10 ng/ml LIF, 10 ng/ml OSM, 500 ng/ml GIL-11 for 15 min. Equal amounts of proteins (50 µg/lane) were analyzed via specific antibodies detecting phospho-STAT3 and STAT3. Western blot data shows one representative experiment out of three.

The proliferation of the murine pre-B cell line Ba/F3 is IL-3 dependent^34^. After the introduction of human gp130 plus additional family receptors (IL-6R, IL-11R^35,36^, OSMR and LIFR (Supplementary Fig. 1B, C)), proliferation shifted to the respective IL-6 type cytokine receptor combination. In case of Ba/F3-gp130 cells, proliferation is induced by IL-11 and the soluble IL-11R or by the corresponding fusion protein Hyper IL-11 (HIL-11)^37^, additional introduction of IL-11R renders these cells IL-11 dependent. Co-expression of gp130 and IL-6R renders these cells responsive to IL-6. Ba/F3 cells expressing gp130 and LIFR proliferate with LIF and OSM, whereas gp130 and OSMR expressing Ba/F3 cells are responsive to OSM. Using our Ba/F3 cell repertoire with the eight different receptor combinations gp130, gp130:IL-11R, gp130:LIFR, gp130:OSMR, gp130:IL-11R:OSMR, gp130:IL-6R:LIFR and gp130:IL-11R:LIFR, we determined the qualitative proliferation properties after addition of IL-11, HIL-11, OSM, LIF and GIL-11. Using a rather high concentration of 500 ng/ml to initially assess possible receptor cross-activation. GIL-11 specifically only induced proliferation of Ba/F3-gp130:IL-11R:LIFR cells but not of any other tested cell line, suggesting that GIL-11 signals via gp130:IL-11R:LIFR (Fig. 2a, Supplementary Data). As expected, only HIL-11 induced proliferation of all Ba/F3 cells lines, since they all express gp130. All Ba/F3 cells expressing gp130 and IL-11R proliferated with IL-11. Expression of gp130 and LIFR resulted in LIF and OSM-induced proliferation, whereas cells expressing gp130 and OSMR cells were OSM selective. Next, we analyzed the phosphorylation of STAT3 which is the major hallmark of IL-6 type cytokine JAK/STAT signaling in our Ba/F3 cell portfolio. As expected from the cytokine-induced Ba/F3 cell proliferation assays (Fig. 2a), STAT3 phosphorylation in Ba/F3 cells was induced by the following cytokine:cytokine receptor combinations: HIL-11 via gp130; IL-11 via gp130:IL-11R; OSM via gp130:OSMR; OSM and LIF via gp130:LIFR (Fig. 2b; Supplementary Figs. 2, 3). Importantly, sustained STAT3 phosphorylation for GIL-11 was only observed in Ba/F3 cells expressing the receptor combination gp130, IL-11R and LIFR (Fig. 2b; Supplementary Figs. 2, 3). Taken together, our data also showed that GIL-11 specifically activated signaling via the non-natural receptor complex consisting of gp130:IL-11R:LIFR.

### GIL-11 transcriptome pattern differs from IL-11 and LIF

**Fig. 3:**
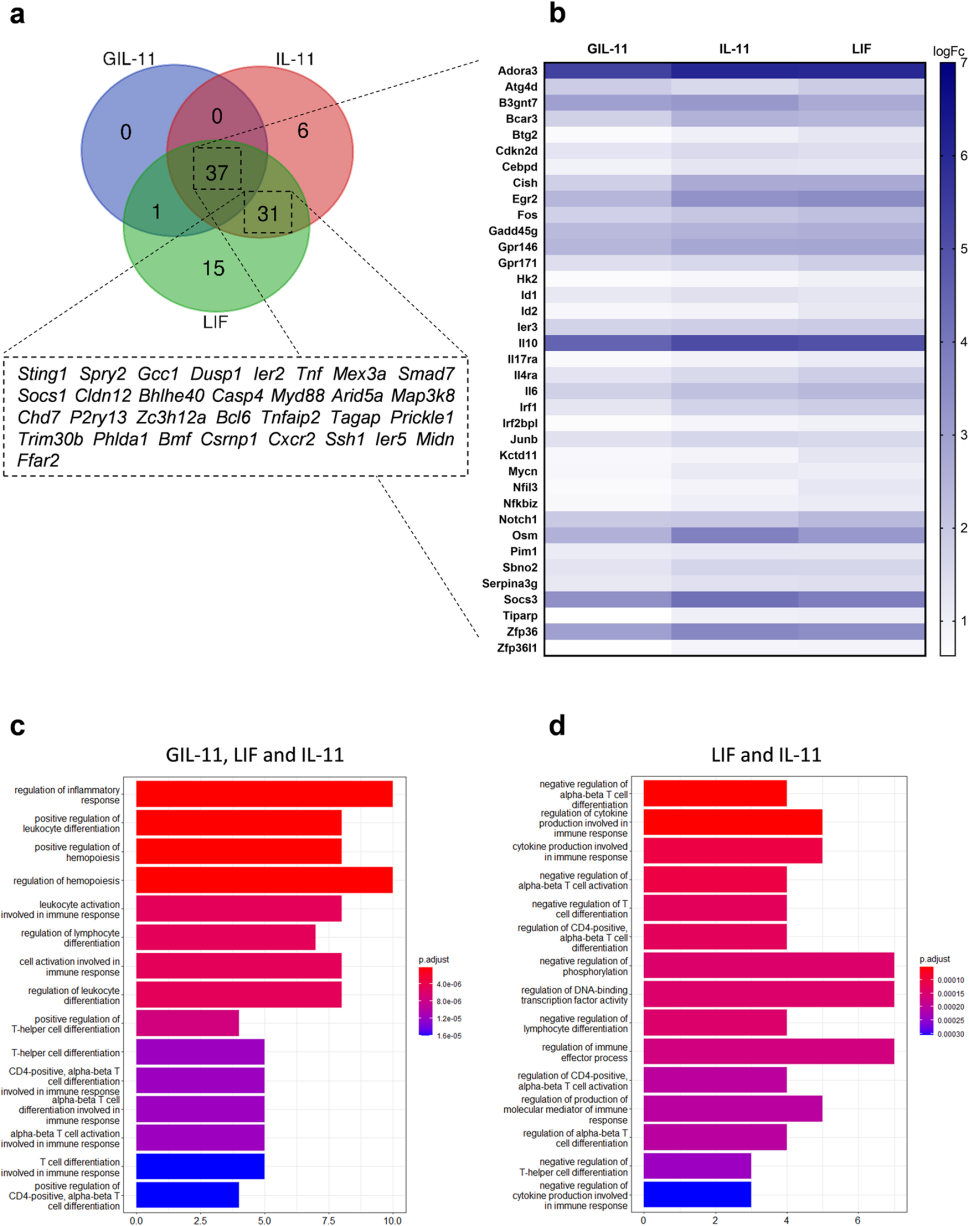
Transcriptome profiling of Ba/F3-gp130:IL-11R:LIFR cells demonstrate attenuated gene activation by GIL-11. **a** Venn Diagram shows overlap of genes that are activated by natural or synthetic cytokines. Filter: *p* < 0.05 including false discovery rate correction; |FC| ≥ 1.5. **b** Heat map shows genes that are significant increased by GIL-11 vs untreated, IL-11 vs untreated and LIF vs untreated. Scale bar shows log fold change. Filter: *p* < 0.05 including false discovery rate correction; |FC| ≥ 1.5 Gene ontology analysis of common gene pathways that are activated by GIL-11, LIF and IL-11 (**c**) and by the natural cytokines only (**d**). Filter: *p* < 0.05 including false discovery rate correction; |FC| ≥ 1.5. X-axis: number of genes involved in common gene pathway. Gene expression accession code: GSE226064.

To evaluate the transcription profile of GIL-11, Ba/F3-gp130:IL-11R:LIFR cells were stimulated for 40 min with GIL-11, IL-11 or LIF with 100-fold EC50 concentrations to ensure maximum signal transduction and comparability for transcriptome analysis. Venn diagram shows 37 genes were at least 1.5-fold upregulated by GIL-11, IL-11 and LIF (Fig. 3a, b). Interestingly, 31 genes were at least 1.5-fold upregulated by IL-11 and LIF but not by GIL-11. In total, 52 genes were upregulated by IL-11 and/or LIF but not by GIL-11 suggesting GIL-11 leads to a more attenuated transcription compared to IL-11 or LIF. Gene ontology enrichment analysis shows that several genes involved in inflammatory responses or hematopoiesis are upregulated by GIL-11, IL-11 and LIF (Fig. 3c, d). Taken together, our data demonstrate the transcription pattern of GIL-11 slightly differs from the natural cytokines IL-11 and LIF.

### Biological activity of GIL-11 is comparable to IL-11 and LIF

**Fig. 4:**
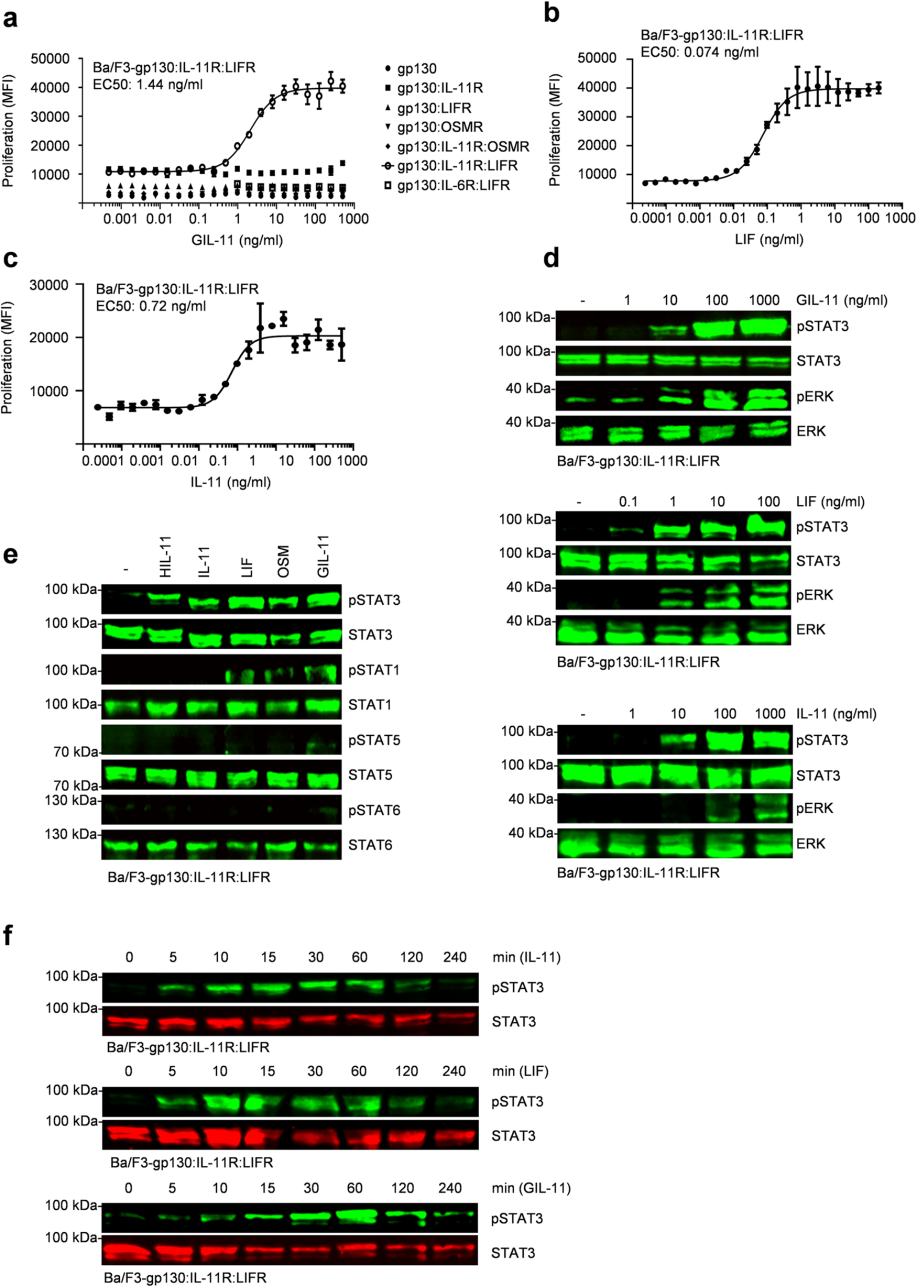
Biological activity of GIL-11 is comparable to IL-11 and LIF. **a** Proliferation of Ba/F3, Ba/F3-gp130, Ba/F3-gp130:IL-11R, Ba/F3-gp130:LIFR, Ba/F3-gp130:OSMR, Ba/F3-gp130:IL-11R:OSMR, Ba/F3-gp130:IL-6R:LIFR, Ba/F3-gp130:LIFR:IL-11R cells in the presence and absence of increasing concentrations of GIL-11 (0.0005–500 ng/ml). **b** Proliferation of Ba/F3-gp130:IL-11R:LIFR cells in the presence and absence of increasing concentrations of LIF (0.000025–200 ng/ml). **c** Proliferation of Ba/F3-gp130:IL-11R:LIFR cells in the presence and absence of increasing concentrations of IL-11 (0.000025–500 ng/ml). **a**–**c** Error bars define ±SEM. One representative experiment with three biological replicates out of three is shown. **d** STAT3 and ERK activation in Ba/F3-gp130:LIFR:IL-11R cells without cytokine (-) and after stimulation with increasing amounts of GIL-11 (1, 10, 100, 1000 ng/ml), LIF (0.1, 1, 10, 100 ng/ml) and IL-11 (1, 10, 100, 1000 ng/ml) for 15 min. Equal amounts of proteins (50 µg/lane) were analyzed via specific antibodies detecting phospho-STAT3, STAT3, ERK and phospho-ERK. Western blot data shows one representative experiment out of three. **e** STAT1,3,5,6 activation in Ba/F3-gp130:IL-11R:LIFR cells without cytokine (-) and after stimulation with 50 ng/ml HIL-11, 50 ng/ml IL-11, 10 ng/ml LIF, 10 ng/ml OSM, 500 ng/ml GIL-11 for 15 min. Equal amounts of proteins (50 µg/lane) were analyzed via specific antibodies detecting phospho-STATs and STATs. Western blot data shows one representative experiment out of three. **f** Time-dependent STAT3 activation of Ba/F3-gp130:IL-11R:LIFR cells with the cytokines IL-11 (200 ng/ml), LIF (20 ng/ml) and GIL-11 (200 ng/ml) for the indicated time. Equal amounts of proteins (50 µg/lane) were analyzed via specific antibodies detecting phospho-STAT3 and STAT3.

Next, we performed dose-dependent proliferation to determine the quality and capacity of GIL-11. Again, GIL-11 did not induce proliferation of Ba/F3 cells expressing gp130, gp130:IL-11R, gp130:LIFR, gp130:OSMR, gp130:IL-11R:OSMR even at the highest applied concentration of 500 ng/ml, whereas the proliferation of Ba/F3-gp130:IL-11R:LIFR cells was clearly dose-dependent with an EC50 of 1.44 ng/ml (Fig. 4a; Supplementary Data). For comparison, we determined the EC50 for LIF and IL-11 to be 0.074 ng/ml and 0.72 ng/ml on Ba/F3-gp130:IL-11R:LIFR cells (Fig. 4b, c; Supplementary Data). To analyze STAT3 and ERK phosphorylation, Ba/F3-gp130:IL-11R:LIFR cells were stimulated with increasing amounts of GIL-11, IL-11 and LIF. Western blotting showed that 10–100 ng/ml of GIL-11 and IL-11 were needed to achieve maximal STAT3 and ERK phosphorylation, whereas the biological activity of LIF was higher with 0.1–1 ng/ml needed for maximal STAT3 phosphorylation (Fig. 4d, Supplementary Fig. 4). With respect to the activation of the other STATs in Ba/F3-gp130:IL-11R:LIFR cells, phosphorylation of STAT1, 3, 5 and 6 was assessed after stimulation with HIL-11, IL-11, OSM, LIF, and GIL-11. Via gp130, all IL-6-type cytokines efficiently activate STAT3, but only to a minor extent STAT1 and STAT5. In the case of LIF and OSM, STAT3 and STAT1, as well as STAT5, activation was observed^38^. Here, all cytokines induced sustained STAT3 phosphorylation. Interestingly, HIL-11 and IL-11 did not induce STAT1 and STAT5 phosphorylation, whereas LIF, OSM, and GIL-11 also induced STAT1 phosphorylation (Fig. 4e, Supplementary Fig. 6; 2c, d). STAT5 phosphorylation was only seen for GIL-11. As expected, none of the cytokines induced STAT6 phosphorylation. Next, we stimulated Ba/F3-gp130:LIFR:IL-11R cells with IL-11, LIF, and GIL-11 for 5–240 min. STAT3 phosphorylation begins 5 min after cytokine stimulation, with a peak after 30–60 min. As typically seen for IL-11 and LIF, STAT3 phosphorylation declines after 120–240 min, which is likely due to SOCS3 upregulation^39^ (compare Fig. 3b, transcriptomic analysis, SOCS3 upregulation by 10.5-fold GIL-11, 18.4-fold IL-11, 14.5-fold LIF), the same regulation of STAT3 phosphorylation was seen for the cytokine GIL-11 (Fig. 4f, Supplementary Fig. 7). Taken together, the activity of GIL-11 is in the same concentration range as the precursor cytokine IL-11, demonstrating that the expansion of the receptor requirement by site III transfer did not affect the overall biological cytokine activity.

### GIL-11:soluble IL-11R complexes are poor inducer of trans-signaling via gp130:LIFR

**Fig. 5:**
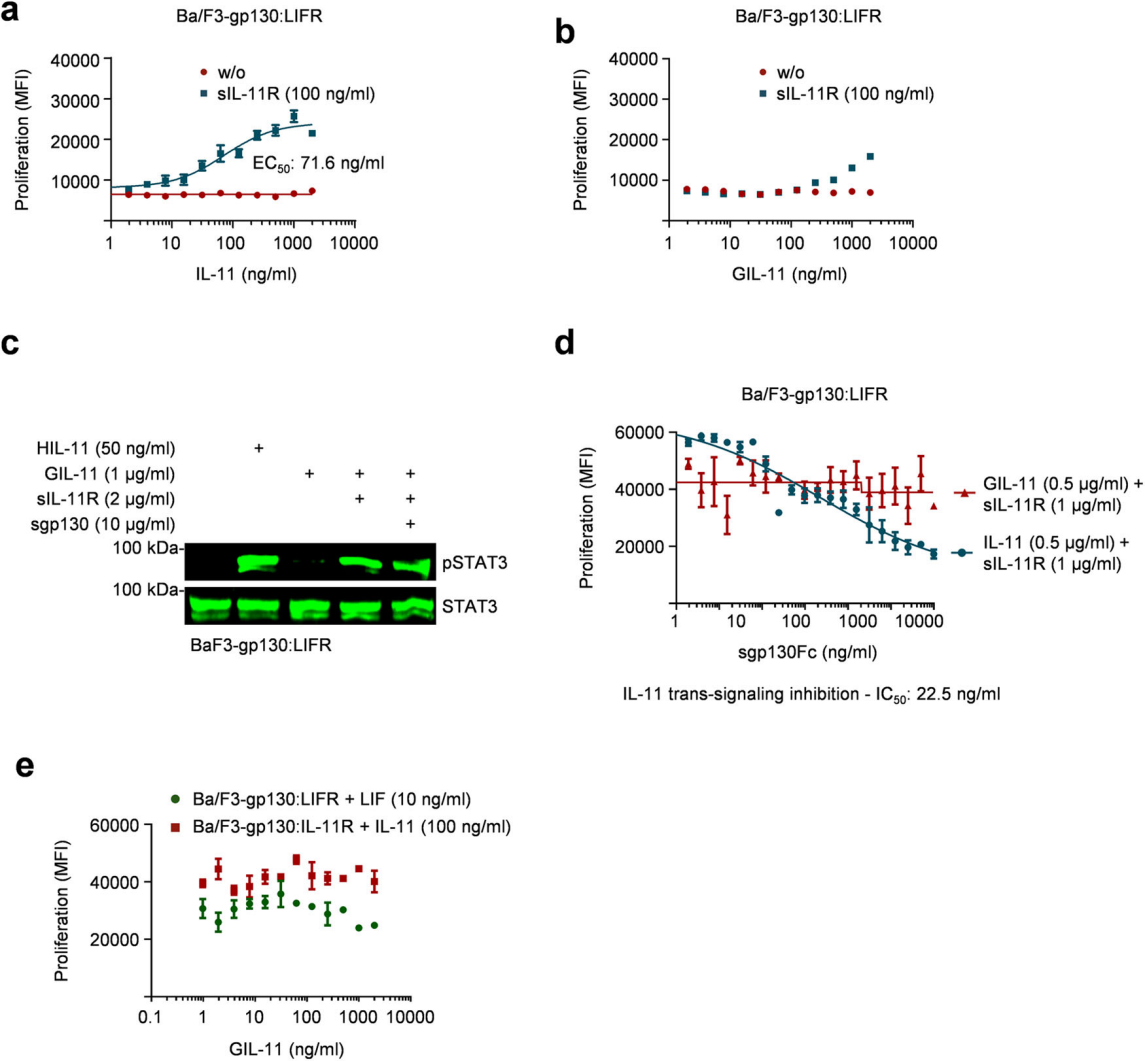
GIL-11:sIL-11 complexes induce trans-signaling via gp130:LIFR. **a** Proliferation of Ba/F3-gp130:LIFR cells in the presence and absence of fixed concentrations of sIL-11R (0 or 100 ng/ml) and increasing concentrations of IL-11 (1–2000 ng/ml). One representative experiment out of three is shown. **b** Proliferation of Ba/F3-gp130:LIFR cells in the presence and absence of fixed concentrations of sIL-11R (0 or 100 ng/ml) and increasing concentrations of GIL-11 (1–2000 ng/ml). One representative experiment out of three is shown. **c** STAT3 activation in Ba/F3-gp130:LIFR cells without cytokine (-) and after stimulation with HIL-11 (50 ng/ml), GIL-11 (1 µg/ml), GIL-11 (1 µg/ml):sIL-11R (2 µg/ml), GIL-11 (1 µg/ml):sIL-11R (2 µg/ml):sgp130Fc (10 µg/ml) for 15 min. Equal amounts of proteins (50 µg/lane) were analyzed via specific antibodies detecting phospho-STAT3 and STAT3. Western blot data shows one representative experiment out of three. **d** Proliferation of Ba/F3-gp130:LIFR cells in the presence and absence of fixed concentrations of IL-11 (0.5 µg/ml):sIL-11R (1 µg/ml) or GIL-11 (0.5 µg/ml):sIL-11R (1 µg/ml) and increasing concentrations of sgp130Fc (1–10,000 ng/ml). One representative experiment out of three is shown. **e** Proliferation of Ba/F3-gp130:LIFR cells in the presence and absence of fixed concentrations of LIF (10 ng/ml) and increasing concentrations of GIL-11 (1–2000 ng/ml) (green). Proliferation of Ba/F3-gp130:IL-11R cells in the presence and absence of fixed concentrations of IL-11 (100 ng/ml) and increasing concentrations of GIL-11 (1–2000 ng/ml) (red). Error bars define ±SEM. One representative experiment with three biological replicates out of three is shown.

The α-receptor dependent cytokines IL-6 and IL-11 activate cells via the signal transducing receptor gp130 and the non-signaling membrane-bound IL-6R or IL-11R, respectively^37,40^. Here, we showed GIL-11 signals via the membrane-bound IL-11R in complex with the heterodimeric gp130:LIFR complex. Signaling via the membrane-bound IL-11R is called classic-signaling. The IL-11R also exists as a soluble receptor that in complex with IL-11 activates cells lacking membrane-bound α-receptor expression in a process called trans-signaling^37,40^. Here, we analyzed to what extent GIL-11 induced trans-signaling in complex with soluble IL-11R (sIL-11R) on cells expressing gp130 and LIFR. Ba/F3-gp130:LIFR cells were stimulated with a fixed concentration of 100 ng/ml sIL-11R and increasing concentrations of GIL-11 (2–2000 ng/ml). For comparison, we used the same fixed concentration of sIL-11R and increasing cytokine concentrations for IL-11. Whereas the EC50 for IL-11 was 71.6 ng/ml for 100 ng/ml sIL-11R (Fig. 5a; Supplementary Data), GIL-11 hardly induced proliferation via sIL-11R. It was not possible to calculate an EC50 value, because even the concentration of 2000 ng/ml GIL-11 was not sufficient to reach maximal cellular proliferation (Fig. 5b; Supplementary Data). Therefore, we evaluated the intracellular signal transduction in Ba/F3-gp130:LIFR cells stimulated with comparably high concentrations of 1 µg/ml GIL-11 and 2 µg/ml sIL-11R. Here, GIL-11:sIL-11R complexes resulted in sustained STAT3 activation. sgp130Fc is a selective IL-6/IL-11 trans-signaling inhibitor, which inhibits LIF signaling at least 100–1000-fold less efficiently than IL-6/IL-11 trans-signaling^40,41^. Next, we tested if sgp130Fc inhibits GIL-11 trans-signaling. As shown in Fig. 5c, Supplementary Fig. 8, sgp130Fc (10 µg/ml) did not inhibit STAT3 phosphorylation induced by GIL-11 (1 µg/ml):sIL-11R (2 µg/ml) trans-signaling in Ba/F3-gp130:LIFR cells (Fig. 5c, Supplementary Fig. 8). For Ba/F3-gp130:LIFR cells, concentrations for GIL-11 (0.5 µg/ml) or IL-11 (0.5 µg/ml) plus sIL-11R (1 µg/ml) were chosen to allow cellular proliferation via trans-signaling. Titration of increasing concentrations of sgp130Fc resulted in the inhibition of IL-11 trans-signaling (IC50 = 22.5 ng/ml, Fig. 5d; Supplementary Data), whereas even the highest concentration of 10 µg/ml sgp130Fc was not able to inhibit GIL-11 trans-signaling. In principle, GIL-11 is able to signal via trans-signaling, albeit with a much lesser efficiency compared to IL-11. Like LIF, GIL-11 trans-signaling is not inhibited by sgp130Fc at least under conditions that are sufficient to block IL-11 trans-signaling^37,40,41^. As shown in Fig. 2a, the proliferation of Ba/F3-gp130:LIFR and Ba/F3-gp130:IL-11R was induced by LIF and IL-11, respectively, but not by GIL-11. We could, however, not exclude that GIL-11 binds to IL-11R:gp130 on Ba/F3-gp130:IL-11R cells and blocks IL-11 signaling or to LIFR on Ba/F3-gp130:LIFR cells and blocks LIF signaling. Using cytokine co-incubation for Ba/F3-gp130:IL-11R with IL-11 and GIL-11 and for Ba/F3-gp130:LIFR with LIF and GIL-11 did not result in inhibition of cellular proliferation even at a 20-fold mass concentration excess of GIL-11 over IL-11 and a 200-fold GIL-11 excess over LIF (Fig. 5e; Supplementary Data). We conclude that GIL-11 did not interfere with IL-11 and LIF signaling at least for the concentration range tested.

### Human GIL-11 activates signal transduction via the murine gp130:IL-11R:LIFR cytokine receptor complex

**Fig. 6:**
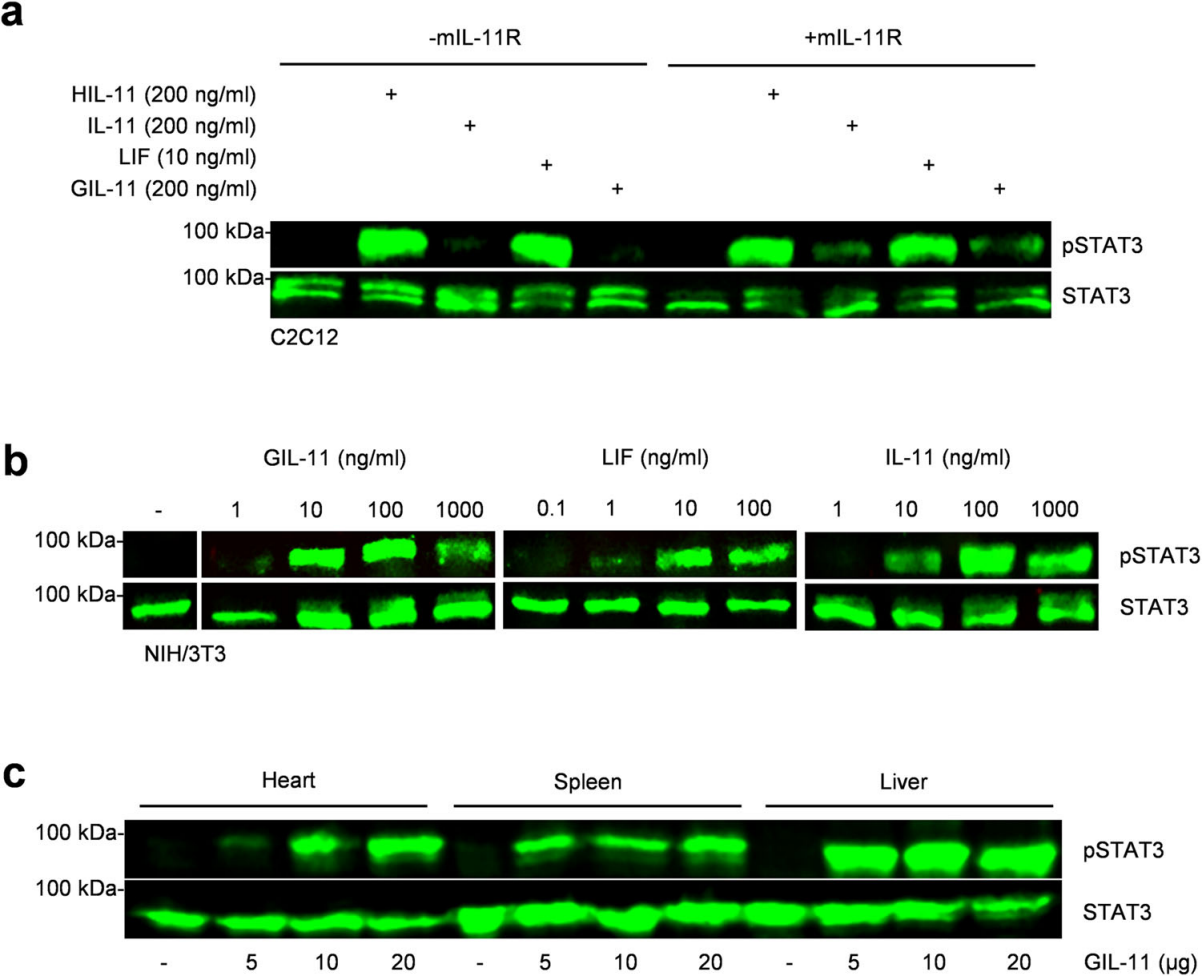
Human GIL-11 transmits signal transduction via the non-natural murine gp130:IL-11R:LIFR cytokine receptor complex. **a** STAT3 activation in un-transfected and transfected with a cDNA coding for murine IL-11R murine myoblasts (C2C12) without cytokine (-) and after stimulation with HIL-11 (200 ng/ml), IL-11 (200 ng/ml), LIF (10 ng/ml), GIL-11 (200 ng/ml) for 15 min. Equal amounts of proteins (50 µg/lane) were analyzed via specific antibodies detecting phospho-STAT3 and STAT3. Western blot data shows one representative experiment out of three. **b** STAT3 activation in murine fibroblast NIH/3T3 without cytokine (-) and after stimulation with increasing amounts of GIL-11 (1, 10, 100, 1000 ng/ml), LIF (0.1, 1, 10, 100 ng/ml) and IL-11 (1, 10, 100, 1000 ng/ml) for 20 min. Equal amounts of proteins (50 µg/lane) were analyzed via specific antibodies detecting phospho-STAT3 and STAT3. Western blot data shows one representative experiment out of three. **c** STAT3 activation in heart, liver and spleen after injection of 5, 10 or 20 µg/ml GIL-11. Mice were sacrificed 30 min after intraperitoneal cytokine injection. Equal amounts of proteins (50 µg/lane) were analyzed via specific antibodies detecting phospho-STAT3 and STAT3. Western blot data shows one representative experiment out of three.

Human IL-11 and LIF are cross-reactive between mice and men^42–44^. We analyzed if human GIL-11 also activates murine cells expressing murine gp130:IL-11R:LIFR chains. We chose the murine myoblast cell line C2C12. C2C12 cells were stimulated with human HIL-11 (200 ng/ml), human IL-11 (200 ng/ml), human LIF (10 ng/ml) and GIL-11 (200 ng/ml). HIL-11 and LIF induced sustained STAT3 phosphorylation whereas IL-11 and GIL-11 did not, suggesting that C2C12 cells express gp130 and LIFR but lack the expression of IL-11R (Fig. 6a, Supplementary Fig. 9). We transfected C2C12 cells with a plasmid coding for murine IL-11R cDNA. Resulting in mIL-11R expression, which makes these cells responsive to IL-11 and GIL-11 as shown by STAT3 phosphorylation (Fig. 6a, Supplementary Fig. 9). We also stimulated murine embryonic fibroblast cells line NIH/3T3 with 1, 10, 100 and 1000 ng/ml human GIL-11, IL-11 and 0.1, 1, 10 and 100 ng/ml LIF. LIF and IL-11 induced STAT3 phosphorylation in NIH/3T3 as shown by Western blotting (Fig. 6b, Supplementary Fig. 10), because these cells express gp130, LIFR and IL-11R^45^. Dose-dependent cellular stimulation showed 10–100 ng/ml of GIL-11 and IL-11 were needed to achieve sustained STAT3 phosphorylation in murine NIH/3T3. Next, we injected 5, 10 and 20 µg GIL-11 intraperitoneally into wild-type mice. 30 min after injection mice were sacrificed and heart, liver and spleen tissue were removed. Analysis of STAT3 phosphorylation by Western blotting showed that at least 10 µg/mouse GIL-11 was sufficient to induce sustained STAT3 phosphorylation in the heart, liver and spleen (Fig. 6c, Supplementary Fig. 11). Taken together, our data demonstrated that human GIL-11 activates the murine receptor combination gp130:IL-11R:LIFR.

### GIL-11 rescued IL-6R^−/−^ mice from death following partial hepatectomy

**Fig. 7:**
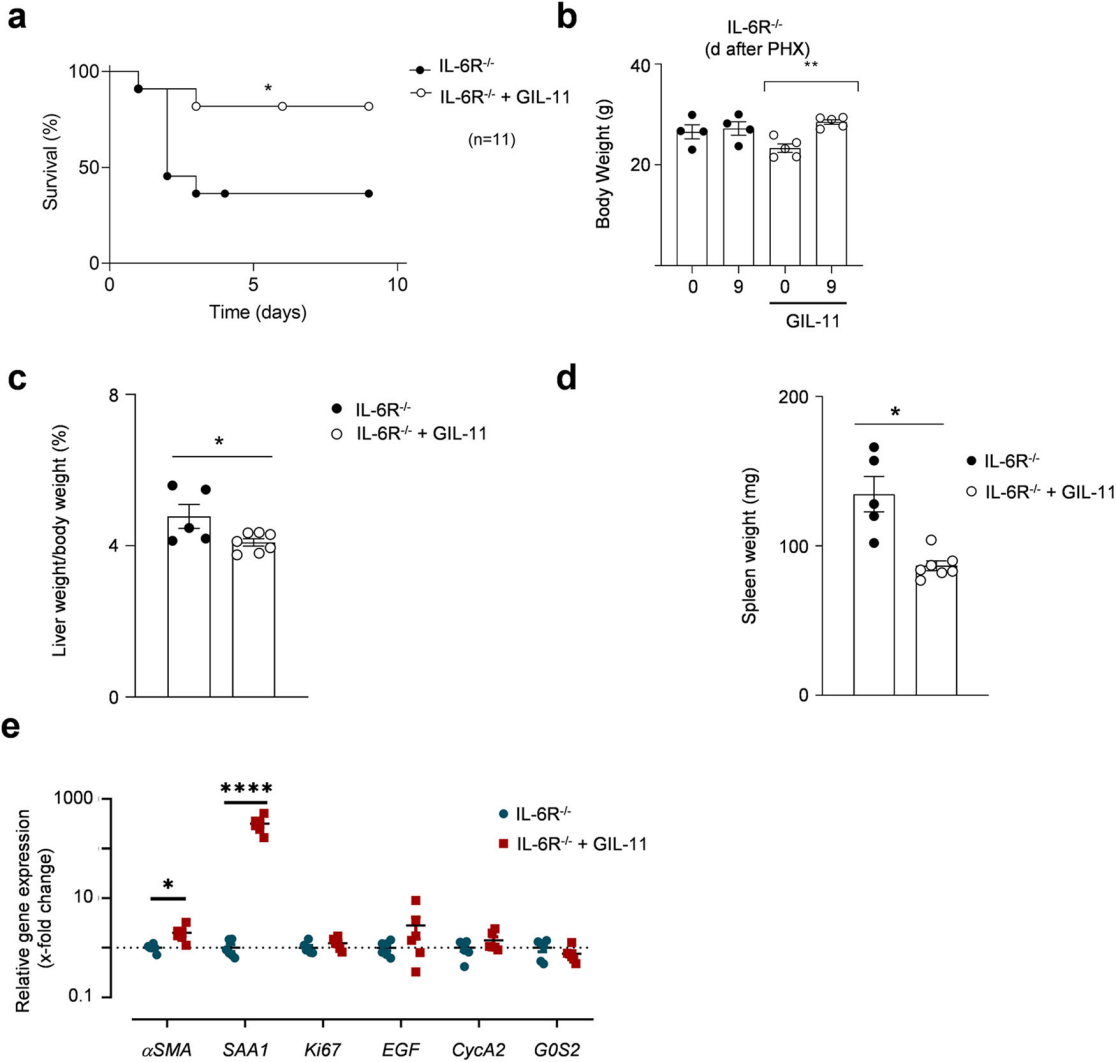
Human GIL-11 rescued IL-6R^−/−^ mice from death following partial hepatectomy. **a** *IL-6R*^*−/−*^ mice were subjected to 70% PHX, injected with PBS (*n* = 11) or GIL-11 (*n* = 11), 20 µg each, 24 h before and directly after surgery and survival was monitored for 9 days. **b** Body weight of *IL-6R*^*−/−*^ mice treated with and without GIL-11 after 70% PHX was determined 12 days after PHX (*n* = 4 untreated, *n* = 5 GIL-11). **c** Liver/body weight ratio of *IL-6R*^*−/−*^ mice treated with and without GIL-11 after 70% PHX was determined at 9 days after PHX (*n* = 5 untreated, *n* = 7 GIL-11). **d** Spleen weight of *IL-6R*^*−/−*^ mice treated with and without GIL-11 after 70% PHX was determined at 9 days after PHX (*n* = 5 untreated, *n* = 7 GIL-11). **e** Total RNA was extracted from the liver directly after PHX from *IL-6R*^*−/−*^ mice previously treated with and without GIL-11 and mRNA level of *aSMA, SAA1, Ki67*, *EGF, CycA2 and G0S2* were determined by quantitative PCR (*n* = 6). **b**–**e** Each dot represent data derived from one mouse. Error bars are ±SEM.

Interleukin-6 (IL-6) is critically involved in liver regeneration following partial hepatectomy (PHX). *IL-6*^*−/−*^ and *IL-6R*^*−/−*^ mice have a high mortality rate of 40–80% versus 10% in wild-type mice accompanied by decreased STAT3 phosphorylation and diminished proliferation of hepatocytes^27,46–50^ followed PHX. We have previously shown that Hyper IL-6 (HIL-6) injection 24 h before and directly after surgery rescued mice from death following partial hepatectomy^51^. Here, 10 µg/mouse GIL-11 was injected 24 h before and directly after PHX in *IL-6R*^*−/−*^ mice. The overall survival rate of *IL-6R*^*−/−*^ mice 9 days after PHX was about 40%, whereas *IL-6R*^*−/−*^ mice injected with two doses of GIL-11 had a survival rate of 90% (Fig. 7a; Supplementary Data). As seen before^27^, body weight 9 days after PHX was not different in surviving *IL-6R*^*−/−*^ mice, irrespective if injected with GIL-11 or PBS, whereas liver weight to body weight ratio was slightly decreased (Fig. 7b, c; Supplementary Data). However, we noticed that untreated *IL-6R*^*−/−*^ mice had a bigger spleen weight compared to GIL-11 treated *IL-6R*^*−/−*^ mice (Fig. 7d; Supplementary Data). Gene expression analysis showed increased expression of fibrotic marker *αSMA* and acute phase response gene *SAA1* in *IL-6R*^*−/−*^ mice treated with GIL-11 compared to untreated *IL-6R*^*−/−*^ mice directly following PHX (Fig. 7e; Supplementary Data). Taken together, our data showed that GIL-11 rescued *IL-6R*^*−/−*^ mice from death following partial hepatectomy.

## Discussion

Our experiments define a human chimeric designer cytokine that induces family-typical JAK/STAT signaling and cellular proliferation via the non-natural gp130:IL-11R:LIFR complex with cross-species specificity from mouse to man. The exchange of site III from LIF to IL-11 results in the cytokimera GIL-11 with receptor binding properties thus far not found in nature. The prototype cytokimera IC7, based on the IL-6 scaffold with site III from CNTF, served as a blueprint for GIL-11^3^. IC7 and GIL-11 revealed that the overall scaffold within the IL-6 type cytokine family is exchangeable due to a general modular architecture. Since the transfer is restricted to site III, it remains to be seen whether the transfer of site I and site II will also be feasible. With the second of its kind, GIL-11 has unique features which separates it from IC7. First of all, IC7 recruits the receptor complex gp130:IL-6R:LIFR, whereas GIL-11 assembles gp130:IL-11R:LIFR, meaning that only those cells expressing IL-11R are targeted by GIL-11. This might be an important issue with respect to cellular specificity in vivo. Whereas the common β-receptor gp130 is ubiquitously expressed, expression of LIFR and also the α-receptors is restricted and therefore more cell type specific. Unlike IL-6R, which in mainly found on immune cells and hepatocytes^52,53^, the distribution of membrane-bound IL-11R appears to be more balanced^54^ including cardiomyocytes^55^, fibroblasts^56^ and epithelial cells^57^.

Due to their limited expression profile, the α-receptors IL-6R, IL-11R and CNTFR confer a second layer of cellular specificity^6^. We hypothesize that the recruitment of synthetic complexes eventually results in a gain-of-function in cellular specificity and thereby might promote beneficially and reduce negative effects during in vivo applications of designer cytokines in synthetic biology. Of note, IC7Fc selectively activates metabolic pathways resulting in increased fatty acid oxidation accompanied by prevented steatosis, increased energy dissipation accompanied by weight loss, muscle hypertrophy and preserved lean mass with improved bone stability in mice^17^. In contrast to IL-6 and CNTF, IC7 injection has proven to be safe in both, mice and non-human primate macaques without promoting excess inflammatory responses^3,17,58^.

Moreover, it was assumed the transcriptomic profile of GIL-11 could cover with LIF, since both recruit and activate the gp130 and LIFR heterodimers, whereas IL-11 recruits gp130 homodimers. Interestingly, GIL-11 acted more in between of IL-11 and LIF but with an attenuated extent. It has been proposed that the geometry and affinity of the ligand-receptor complex may account for functional diversity of particular IL-6 type cytokines^59^. The lack of GIL-11s ability for some gene expression might be beneficial. *Smad7* is a negative regulator of TGF-ß signaling and seems to be involved in pathogenesis of inflammatory bowel diseases (IBDs), including Crohn’s disease (CD) and ulcerative colitis (UC) and mediates intestinal inflammation^60^. Overexpression of *MyD88* has been showed to decrease cardiac function and contributes to cardiovascular autoimmune diseases^61–63^. *Arid5a* is assumed to contribute to cytokine storm. Apart from that *Arid5a*^*−/−*^ *mice* are refractory to endotoxin shock, bleomycin-induced lung injury, and inflammatory autoimmune disease^64^.

Secondly, the natural cytokines IC7, IL-6 and CNTF are dependent on α-receptor binding before binding to gp130 and LIFR^3^, meaning that shaping of the CNTF-derived binding site III in IC7 is a direct consequence of IL-6R binding, as after binding of CNTF to CNTFR. This situation is different for GIL-11. Here, we introduced the α-receptor independent binding site III from LIF into the α-receptor dependent IL-11. As shown here, GIL-11 activates the gp130:LIFR receptor complex only after binding to the non-signal transducing IL-11R. Moreover, GIL-11 was not able to inhibit LIF signaling on Ba/F3-gp130:LIFR cells, which should have been the case, if GIL-11 can bind to LIFR in the absence of the IL-11R. Taken together our experiments showed that albeit the original binding site context of LIF-site III to LIFR is α-receptor independent, re-formatting of LIF-site III into the IL-11 scaffold makes the LIF-site III binding α-receptor dependent. Therefore, not the exact binding site III amino acid composition but rather the interconnection mediated by α-helical shifts of site I with site III defines whether a cytokine is α-receptor dependent or independent^65^.

With respect to biological activity, GIL-11 (EC50: 1.44 ng/ml) is comparable to IL-11 (EC50: 0.72 ng/ml) but less effective than LIF (EC50: 0.074 ng/ml). This might be based on the dominant scaffold effect of IL-11 rather than the minor site III exchange effect from LIF. Biological activity of GIL-11 might, however, be increased by site I D186A mutation, since this amino acid exchange is known to increase the affinity of IL-11 to IL-11R^66^. Improving cytokine binding to the α-receptor is a common strategy to improve overall activity in this cytokine family, as has also been shown for CNTF to CNTFR^67^ and IL-6 to IL-6R^68^.

After having characterized the biological activity and the unique receptor composition of GIL-11, we have investigated the ability of GIL-11 to functionally substitute IL-6 during liver regeneration following PHX in *IL-6R*^*−/−*^ mice. We and others have previously shown that IL-6 and IL-6R are critically involved in liver regeneration after PHX, resulting in higher mortality in *IL-6R*^*−/−*^ mice^27,46–50^. Importantly, HIL-6 rescued mice from death following partial hepatectomy^51^. Moreover, in wild-type mice the combined injection of IL-6 and soluble IL-6R (sIL-6R), but not of IL-6 alone, accelerates liver regeneration after PHX^69^. Likely because hepatocytes express much more gp130 than IL-6R, the increased presence of IL-6 and sIL-6R result in more gp130 activation and stronger IL-6 signaling compared to IL-6 alone^70^. Finally, blockade of IL-6 trans-signaling by sgp130Fc results in increased mortality following PHX^27^. Mechanistically, IL-6 trans-signaling induced hepatocyte growth factor (HGF) production by hepatic stellate cells directly contributed to liver regeneration following PHX^27^. Since, the *IL-6R*^*−/−*^ mice used in this work lack in the expression of IL-6R, both classic- and trans-signaling are disabled. Thus, they exhibit a mortality rate of 90% versus 10% in wild-type mice^27^. A crucial difference between GIL-11 treatment and HIL-6 is that GIL-11 targets only cells expressing gp130:IL-11R:LIFR, which limits the scope of potential targeted cells in the body, whereas HIL-6 targets almost all cells, because unlike IL-11R and LIFR, gp130 is considered to be ubiquitously expressed^71^. However, since IL-11R, LIFR as well as gp130 are expressed on hepatocytes, GIL-11 was able to compensate for IL-6 trans-signaling and rescued *IL-*6R^−/−^ mice from death following PHX. Interestingly, most parameters including body, liver and spleen weight were not changed in surviving GIL-11-treated and untreated mice. GIL-11, however, increases *SAA1* more than 300-fold even nine days following PHX. *SAA1* induces the proliferation of hepatic stellate cells^72^, which might contribute to liver regeneration following GIL-11 application.

Having shown the general in vitro and in vivo activity, we believe that GIL-11 will be of common interest for conditions in which IL-6 type cytokines including IC7 show beneficial effects^73^. Recently, the originally described beneficial effects of human IL-11^6^ in murine models of human diseases has been challenged. It was stated that the mode of action of injected human IL-11 into mice actually relies on competitive inhibition of endogenous IL-11 signaling^26^. It was concluded that IL-11 has rather detrimental than beneficial effects in a variety of murine disease models, including non-alcoholic steatohepatitis, cardiovascular fibrosis, idiopathic pulmonary fibrosis and fibrotic lung disease^26^. Interestingly, IL-11 was considered to preferentially induce ERK signaling and not, like IL-6, which acts via the same gp130 homodimer, STAT3 phosphorylation^26^. Therefore, we treated murine myoblasts (C2C12 cells) with recombinant HIL-11, IL-11 and GIL-11 in the presence and absence of murine IL-11R, which, however, resulted in sustained IL-11R-dependent STAT3 phosphorylation. Injection of GIL-11 into mice also resulted in sustained STAT3 phosphorylation in heart, liver and spleen tissue, demonstrating that our human cytokines and GIL-11 activate canonical gp130 and gp130:LIFR signaling pathways characterized by STAT3 phosphorylation. For unknown reasons and unlike IL-11, GIL-11 is only poorly inducing trans-signaling via GIL-11:sIL-11R complexes and is not inhibited by sgp130Fc. It will be interesting to see, what will be the consequence of GIL-11 application for murine models of fibrotic diseases.

Although LIF is related to fertility and a series of neurological disorders including multiple sclerosis, recombinant LIF is not used as therapeutic^74,75^. GIL-11s ability to induce LIF-like signaling via gp130:LIFR complexes might open LIF-like applications as a as potential surrogate for recombinant hLIF. Since GIL-11 activity need cells not only expressing LIFR but also the IL-11R, GIL-11s activity is restricted to a lower limited number of target cells compared to LIF which might alleviate potential unwanted negative side effects.

In conclusion, our study defines GIL-11 as a to the best of our knowledge novel promising cytokimera with specific high-affinity activation of the non-natural receptor gp130:LIFR:IL-11R complex. The modular architecture of cytokimeras in general enables a wide range of targeted receptor combinations and directed cell targeting.

## Methods

### Cloning

The cDNA coding for GIL-11 was ordered by BioCat GmbH, which was codon optimized and based on human IL-11 and LIF. The GIL-11 cDNA was then inserted into pcDNA3.1 expression vector including 5′ signal peptide for human IL-11R (Q14626, aa 1–24) followed by sequences for myc tag (EQKLISEEDL) and the fragment encoding for GIL-11, Gly4Ser linker, a TEV recognition site and a twin-strep-tag.

### Molecular modeling

Protein models were generated via the Phyre2 web portal^76^. Complex models and structure-based sequence alignments were generated using UCSF Chimera version 1.13.1, developed by the Resource for Biocomputing, Visualization, and Informatics at the University of California, San Francisco, with support from NIH P41-GM103311^77^.

### Cells, reagents and recombinant proteins

The generation of Ba/F3-gp130, Ba/F3-gp130:IL-6R and Ba/F3-gp130:IL-11R cells was described elsewhere^35,36^. The packaging cell line Phoenix-Eco was received from Ursula Klingmüller (DKFZ, Heidelberg, Germany). NIH/3T3 cells were purchased from the Leibnitz Institute DSMZ-German Collection of Microorganisms and Cell Culture (Braunschweig, Germany). All cells were grown at 37 °C with 5% CO_2_ in a water-saturated atmosphere in Dulbecco’s modified Eagle’s medium (DMEM) high-glucose culture medium (GIBCO^®^, Life Technologies, Darmstadt, Germany) with 10% fetal calf serum (GIBCO®, Life Technologies) and 60 mg/l penicillin and 100 mg/l streptomycin (Genaxxon Bioscience GmbH, Ulm, Germany). Murine Ba/F3-gp130 cells were obtained from Immunex (Seattle, WA, USA) and grown in the presence of HIL-6. 0.2% (10 ng/ml) conditioned medium from a stable clone of CHO-K1 cells secreting HIL-6 in the supernatant^78^. Expi-293F™ cells (ThermoFisher Scientific) were cultured in Expi293™ expression medium without antibiotics until they reached a density of 3–5 × 10^6^ c/ml in a 37 °C incubator with 8% CO_2_ on an orbital shaker at 125 rpm. Synthetic ligands were expressed and purified as described^79^. Recombinant human OSM (catalog no. 295-OM) and recombinant human LIF (catalog no. 7734-LF) were purchased from R&D Systems (Minneapolis, MN, USA). An expression plasmid for IL-11 pET22-IL-11-His6 was used for the expression of IL-11. IL-11 was expressed as a soluble protein in *Escherichia coli* and purified via immobilized metal affinity chromatography. Sgp130FC was expressed in ExpiCHO^TM^ cells (ThermoFisher Scientific). Cells were cultured in ExpiCHO^TM^ Medium and transfected according to vendor manuel (Catalog Number: A29133). The protein was purified as previous described^80^.

### Stimulation assay

Ba/F3-gp130 cell lines were washed three times with PBS to remove cytokines and starved in serum-free DMEM for 3 h. Inhibitor sgp130Fc or sIL-11R were added 5 min prior to stimulation. Cells were stimulated for 15 min (or as indicated) with purified protein (concentration as indicated), harvested, frozen in liquid nitrogen and then lysed. In case of the C2C12 and NIH/3T3 cells, the cells were washed after the stimulation with PBS once before they were detached by 0.05% trypsin, 0.1% EDTA (Genaxxon, catalog. C4261.0100) treatment for 5 min and washed again. Cells were lysed for 45 min with buffer containing 10 mM Tris-HCl, pH 7.5, 150 mM NaCl, 0.5 mM MgCl_2_ and a cOmplete, EDTA-free protease inhibitor mixture tablet (Roche Diagnostics, Mannheim, Germany). Protein concentration was determined by a BCA protein assay (Thermo Fisher Scientific) according to the manufacturer’s instruction. Protein expression and pathway activation was then analyzed by western blotting.

### Western blotting

Fifty micrograms total protein were loaded each lane and separated by SDS-PAGE under reducing conditions and transferred to a nitrocellulose membrane (Amersham Protan; Cytiva; LC, UK; catalog no. 10600016). Blocking of membrane was performed with blocking buffer (Intercept® Blocking Buffer; LI-COR; USA; catalog no. 927-60001) diluted 1:3 in TBS (10 mM Tris-HCl pH 7.6, 150 mM NaCl) for 1 h. Primary antibodies (Phospho-STAT3; Tyr-705; D3A7; catalog no. 9145, STAT3; 124H6; catalog no. 9139, Erk1/2; L34F12; catalog no. 4696, Phospho-Erk1/2; D13.14.4E; catalog no. 4370, Cell Signaling Technology, USA were diluted 1:1000 in blocking buffer containing 0.2% Tween-20 (Sigma-Aldrich; USA; catalog no. P1379-1L) for at least 90 min at ambient temperature or overnight at 4 °C. Membranes were washed with TBS-T (0.1% Tween-20) and then incubated with secondary fluorophore-conjugated antibodies 1:10,000 (IRDye^®^ 800CW Donkey anti-Rabbit; catalog no. 926-32213 and IRDye^®^ 680RD Donkey anti-Mouse; catalog no. 926-68072, LI-COR; USA) for 1 h. Signal detection was achieved using LI-COR Odyssey; USA; Model 2800). Secondary antibodies were detected simultaneously on different channels. Data analysis was conducted using Image Studio Lite 5.2. Liver, spleen and heart tissue were lysed in lysis buffer (50 mM Tris-HCl pH 7.5, 150 mM NaCl, 2 mM EDTA pH 8.0, 2 mM NaF, 1 mM Na_3_VO_4_, 1% NP-40, 1% Triton X-100, 1 cOmplete protease inhibitor cocktail tablet). After lysis, the protein content was measured by BCA assay. Fifty micrograms total protein amount was then loaded each line followed by Immunoblotting. Antibodies used for blotting of lysed animal organs were as follows: anti-p-STAT3 (catalog no. 9145), anti-total-STAT3 (catalog no. 9139).

### Cell viability assay

Ba/F3-gp130 cell lines were washed three times with PBS to remove cytokines from the medium. Cells with a density of 5 × 10^4^ cells/ml were suspended in DMEM containing 10% fetal calf serum, 60 mg/l penicillin and 100 mg/ml streptomycin. Cells were cultured for 3 days in a volume of 100 µl with or without cytokines or inhibitor in the indicated concentrations. The CellTiter Blue Viability Assay (Promega, Karlsruhe, Germany) was used to determine the approximate number of viable cells by measuring the fluorescence (λ_ex_560 nm/λ_em_590 nm) using the Infinite M200 Pro plate reader (Tecan, Crailsheim, Germany). After adding 20 µl/well of CellTiter Blue reagent (time point 0), fluorescence was measured after 60 min every 20 min for up to 2 h. For each condition of an experiment, 3 wells were measured. All values were normalized by subtracting time point 0 values from the final measurement.

### Transfection of cells

Ba/F3-gp130 cell lines were retrovirally transduced with the pMOWS expression plasmids as described^34^. Transduced cells were grown in DMEM medium as described above supplemented with 10 ng/ml HIL-6. Selection of transduced Ba/F3-gp130 cells was performed with puromycin (1.5 μg/ml) or hygromycin B (1 mg/ml) (Carl Roth, Karlsruhe, Germany) for at least 2 weeks. Afterwards, the generated Ba/F3-gp130 cell lines were analyzed for receptor cell surface expression via flow cytometry. C2C12 cells were transfected by 7.5 µg pcDNA3.1 plamid encoding for mIL-11R cDNA and 15 µl TurboFect and incubated for 48 h.

### Cell surface detection of cytokine receptors via flow cytometry

Cell surface expression of stably transfected Ba/F3-gp130 cell lines was detected by specific antibodies. 5 × 10^5^ cells were washed in FACS buffer (PBS, 1% BSA) and then incubated in 50 µl of FACS buffer containing the indicated specific primary antibody (anti-LIFR or –OSMR; 1:20; catalog no. BAF249 and BAF4389, R&D Systems; MN, USA). After incubation of at least 1 h at room temperature, cells were washed and resuspended in 50 µl of FACS buffer containing secondary antibody (NothernLights 493-conjugated anti-goat IgG 1:200) and incubated for 30 min at room temperature. Cells were washed and resuspended in 500 µl of FACS buffer and analyzed by flow cytometry (BD FACSCanto II flow cytometer using the FACSDiva software, BD Biosciences). Data analysis was conducted using FlowJo Version 10 (Tree Star Inc, USA).

### Animals and ethics statement

C57BL/6 and *IL-6R*^*−/−*^ mice^52^ were obtained from the Jackson Laboratory and the animal facility of the Heinrich-Heine University of Düsseldorf, respectively. The experiments of this study were carried out according to the requirements of LANUV-NRW, Germany with the approval number 84-02.04.2019.A303.

### 3′-RNA-Seq analyses

Ba/F3-gp130-IL-11R:LIFR cells were stimulated with GIL-11, IL-11 or LIF for 40 min at 37 °C. mRNA was isolated with NucleoSpin RNA (Macherey-Nagel, Düren, Germany; cat. no. 740955.250) according to vendor’s manual. DNase digested total RNA samples used for 3′-RNA-Seq analyses were quantified (Qubit RNA HS Assay, Thermo Fisher Scientific) and quality measured by capillary electrophoresis using the Fragment Analyzer and the ‘Total RNA standard Sensitivity Assay’ (Agilent Technologies, Inc. Santa Clara, USA). All samples in this study showed very high quality RNA Quality Numbers (RQN; mean = 10.0). The library preparation was performed according to the manufacturer’s protocol using the QuantSeq 3′ mRNA-Seq Library Prep Kit FWD from Lexogen®. Input mount was 200 ng total RNA. Bead purified libraries were normalized and finally sequenced on the NextSeq2000 system (Illumina Inc. San Diego, USA) with a read setup of SR 1 × 100 bp. The Illumina DRAGEN FASTQ Generation tool (version 3.8.4) was used to convert the bcl files to fastq files as well for adapter trimming and demultiplexing. The gene ontology analysis was performed with the r package clusterprofiler and r version 4.1.3.

### Animals

All mice were kept under specific pathogen-free conditions and handled according to regulations defined by FELASA and the national animal welfare body GV-SOLAS (www.gv-solas.de). All transgenic animals were on C57BL/6 N background. Mice were fed with a standard laboratory diet and given autoclaved tap water *ad libitum*. They were kept in an air-conditioned room with controlled temperature (20–24 °C), humidity (45–65%), and day/night cycle (12 h light, 12 h dark). Laparotomy was performed predominantly on male mice at least at 10-12 weeks of age using isoflurane inhalation narcosis 1.5–2% isoflurane with 1 l/min oxygen^81^. In order to perform 70% partial hepatectomy, the right upper lobe, left upper lobe and left lower lobe of liver together with the gallbladder was resected via one-step ligature using 5-0 polyester suture tie (B. Braun Surgical, S.A., Rubi, Spain). Thereafter, the abdominal cavity and outer layer of skin was closed by 5-0 polyglycolic acid (HR13, B. Braun Surgical, S.A., Rubi, Spain) and 4-0 polypropylene monofilament (DS16, B. Braun Surgical, S.A., Rubi, Spain) respectively. In order to reduce the mild pain from operation mice were treated with 5 mg/kg Carprofen (Rimadyl; Pfizer, Wurselen, Germany) after surgery. *IL-6R*^*−/−*^ mice were subjected to 70% partial hepatectomy. At specific time points (0, 12, and 24 h) after surgery mice were weighed and anesthetized (100 mg/kg ketamine, 10 mg/kg xylazine; Vetoquinol GmbH, Ravensburg, Germany). Upon anesthesia mice were bled in order to generate the serum for further analysis. For the liver tissue, liver was rinsed with phosphate-buffered saline (PBS) and weighed to calculate liver weight to body weight ratio and tissue samples were stored at −80 °C for histology and RNA and protein extraction.

### GIL-11 expression, purification and injection into mice

GIL-11 was produced and secreted by Expi293 cells (Thermo Fisher) and purified by Strep-Tag affinity chromatography (Strep-TactinXT 4flow; IBA, catalog no. 2-5023-001) according to the manufacturer’s manual. In order to force cytokine signaling, mice were injected intraperitoneal (i.p.) with 20 µg GIL-11 24 h before and directly after surgery.

### Gene expression analysis

Total RNA was extracted from liver and spleen using Trizol (Thermo Fisher Scientific, Waltham, MA, USA). RNA concentration was measured with NanoDrop 2000c spectrophotometer (Thermo Scientific, Waltham, MA, USA, cat. #172-5140) and adjusted to 100 ng/µl for all samples. To determine the expression of specific genes, iTaq^TM^ Universal SYBR green One-Step Kit (BioRad, California, USA, catalog no. 1725151) was used. Master Mix was prepared according to the manufacturer’s instructions. 5 µl of iTaq universal probe reaction mix (2×), 0.125 µl of iScript advanced reverse transcriptase, 0.125 µl of primers and 200 ng of RNA was used. The total volume of the mixture was then adjusted to 10 µl by adding Nuclease-free H_2_O. For analysis, the expression levels of all target genes were normalized to glyceraldehyde 3-phosphate dehydrogenase (*gapdh*) expression (ΔCT). Gene expression values were the calculated based on the ΔΔCt method. Relative quantities^82^ were determined using the equation: RQ = 2^−ΔΔCt^. The expression level of target genes was determined by ABI 7500 real-time PCR System (Thermo Fisher Scientific, Waltham, MA, USA). The following primer pairs were used in this study: *GAPDH fw* 5′ TCCCACTCTTCCACCTTCGA, *GAPDH rev* 5′ AGTTGGGATAGGGCCTCTCTT, *SAA1 fw* 5′ GACACCATTGCTGAGCAGGAA, *SAA1 fw* 5′ GGGAGTCCAGGAGCTCTGTAG, *Ki67 fw* 5′ GCCGAGTCTGGCATTGAA, *Ki67 rev* 5′ TTTTCTTTCTTCTTTTGCTGAGG, EGF fw 5′ TTCTCACAAGGAAAGAGCATCTC, EGF rev 5′ GTCCTGTCCCGTTAAGGAAAAC, Cyclin A2 fw 5′ GAGGTGGGAGAAGAATATAA, Cyclin A2 rev 5′ ACTAGGTGCTCCATTCTCAG, G0S2 fw 5′ TCTCTTCCCACTGCACCCTA, G0S2 rev 5′ TCCTGCACACTTTCCATCTG, *aSMA fw* 5′ CTGACAGAGGCACCACTGAA, *aSMA rev* 5′ CATCTCCAGAGTCCAGCACA.

### Statistics and reproducibility

#### Statistical analysis

Data are provided as arithmetic means ± SEM using GraphPad Prism, Version 8. Statistically significant differences between two groups were determined with a Students’s *t*-test, including Welch’s correction if indicated. Statistically analysis between several groups were determined using a two-way ANOVA, including Tukey or Dunnet’s correction. Significance was calculated as follows *p* > 0.05: n.s.; *p* < 0.05: *; *p* < 0.01: **; *p* < 0.001: ***; *p* < 0.0001: ****. In vitro assays were performed at least in three independent experiments. For in vivo experiments littermate mice were used as independent individual specimens.

#### Statistical analysis of RNA-Seq

Data analyses on fastq files were conducted with CLC Genomics Workbench (version 22.0.2, QIAGEN, Venlo. NL). After UMI (Unique Molecular Identifier) filtering, all remaining reads of all probes were adapter trimmed and quality trimmed (using the default parameters: bases below Q13 were trimmed from the end of the reads, ambiguous nucleotides maximal 2). Mapping was done against the *Mus musculus* (mm39; GRCm39.107) (July 20, 2022) genome sequence. After grouping of samples (for biological replicates each) according to their respective experimental condition, the statistical differential expression was determined using the Differential Expression for RNA-Seq tool (version 2.7). The Resulting *P* values were corrected for multiple testing by FDR correction. A *P* value of ≤0.05 was considered significant. The Gene Set Enrichment Test (version 1.2) was done with default parameters and based on the GO term ‘biological process’ (*M. musculus*; December 16, 2021). For each group *n* = 4 biologically independent samples were used.

### Reporting summary

Further information on research design is available in the Nature Portfolio Reporting Summary linked to this article.

## Supplementary information

Supplemental Material

Description of Additional Supplementary Files

Supplementary Data

Reporting Summary

## Data Availability

The authors declare that the data supporting the findings of this study are available within the manuscript and from the authors on request. Gene expression data of 3′-RNA-Seq are available at NCBI Gene Expression Omnibus; accession code: GSE226064. The plasmid coding for GIL-11 has been deposited at Addgene; Plasmid ID: 199627 and will be provided upon request.
