# Supplementary material for: Cytokimera GIL-11 rescued IL-6R deficient mice from partial hepatectomy-induced death by signaling via non-natural gp130:LIFR:IL-11R complexes
Source: Commun Biol. 2023 Apr 15;6:418. doi: 10.1038/s42003-023-04768-4 (PMC10105715; doi:10.1038/s42003-023-04768-4)
Supplement: Supplementary file 1 — Supplemental Material [file 42003_2023_4768_MOESM1_ESM.pdf]

Supplementary Figure 1

a

```
hLIF                                                    ILYYTAQGEPFPNNLDKLCGPNVT
GIL-11      -GPPPGPPRVSPDPRAELDSTVLLTRSLLADTRQLAILYYTAQGEPFPNNLDKLCGPNVT        59
hIL-11      PGPPPGPPRVSPDPRAELDSTVLLTRSLLADTRQLAAQLR----DKFPA-----DGDHNL        51
            ***********************************        : **       * :

hLIF        D                                     ITRDQKILNPSALSLHSKLN
GIL-11      DSLPTLAMSAGALGALQLPGVLTRLRADLLSYLRHVQWITRDQKILNPSALSLHSKLNTL        119
hIL-11      DSLPTLAMSAGALGALQLPGVLTRLRADLLSYLRHVQWLRRAGG---SSLKTLEPELGTL        108
            **************************************: *       *  :*. :*.**

hILF                                     TSGKDVFQKKKLGCQLLG
GIL-11      QARLDRLLRRLQLLMSRLALPQPPPDPPAPPLATSGKDVFQKKKLGCQLLGGLHLTLDWA        179
hIL-11      QARLDRLLRRLQLLMSRLALPQPPPDPPAPPLAPPSS-AWGGIRAAHAILGGLHLTLDWA        167
            *********************************  .. .:   : .  :***********

hLIF
GIL-11      VRGLLLLKTRL 190
hIL-11      VRGLLLLKTRL 178
            ***********
```

b

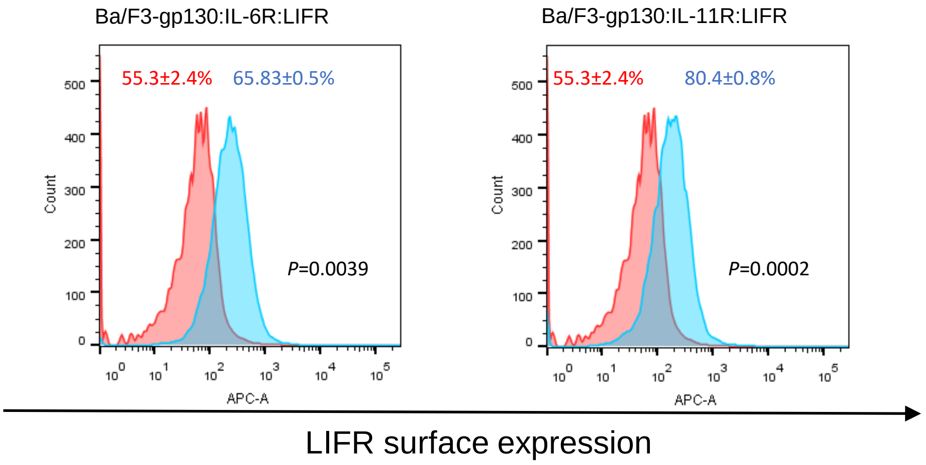

c

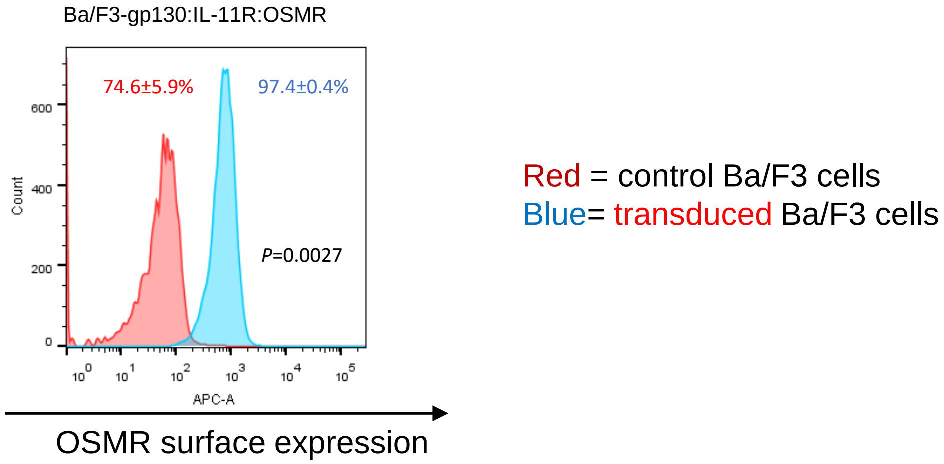

Supplemental Figure 1. **(a)** Amino acid sequence alignment of hLIF, GIL-11 and hIL-11. **(b)** Cell surface expression of Ba/F3-gp130:IL-6R and Ba/F3-gp130:IL-11R cells transduced with human LIFR and/or OSMR (blue shades). Expression was demonstrated via receptor antibodies. Red-shade areas indicate Ba/F3-gp130:IL-6R cells (negative control). Depicted is one flow cytometry experiment out of three. Populations in a gate is mean ±SEM. *P*-value was determined by Dunnett-test with untransduced group as the control group. **(c)** Cell surface expression of Ba/F3-gp130:IL-11R cells transduced with human OSMR (blue shades). Red-shade areas indicate Ba/F3-gp130:IL-11R cells (negative control). Depicted is one flow cytometry experiment out of three. Populations in a gate is mean ±SEM. *P*-value was determined by student t-test.

Supplementary Figure 2

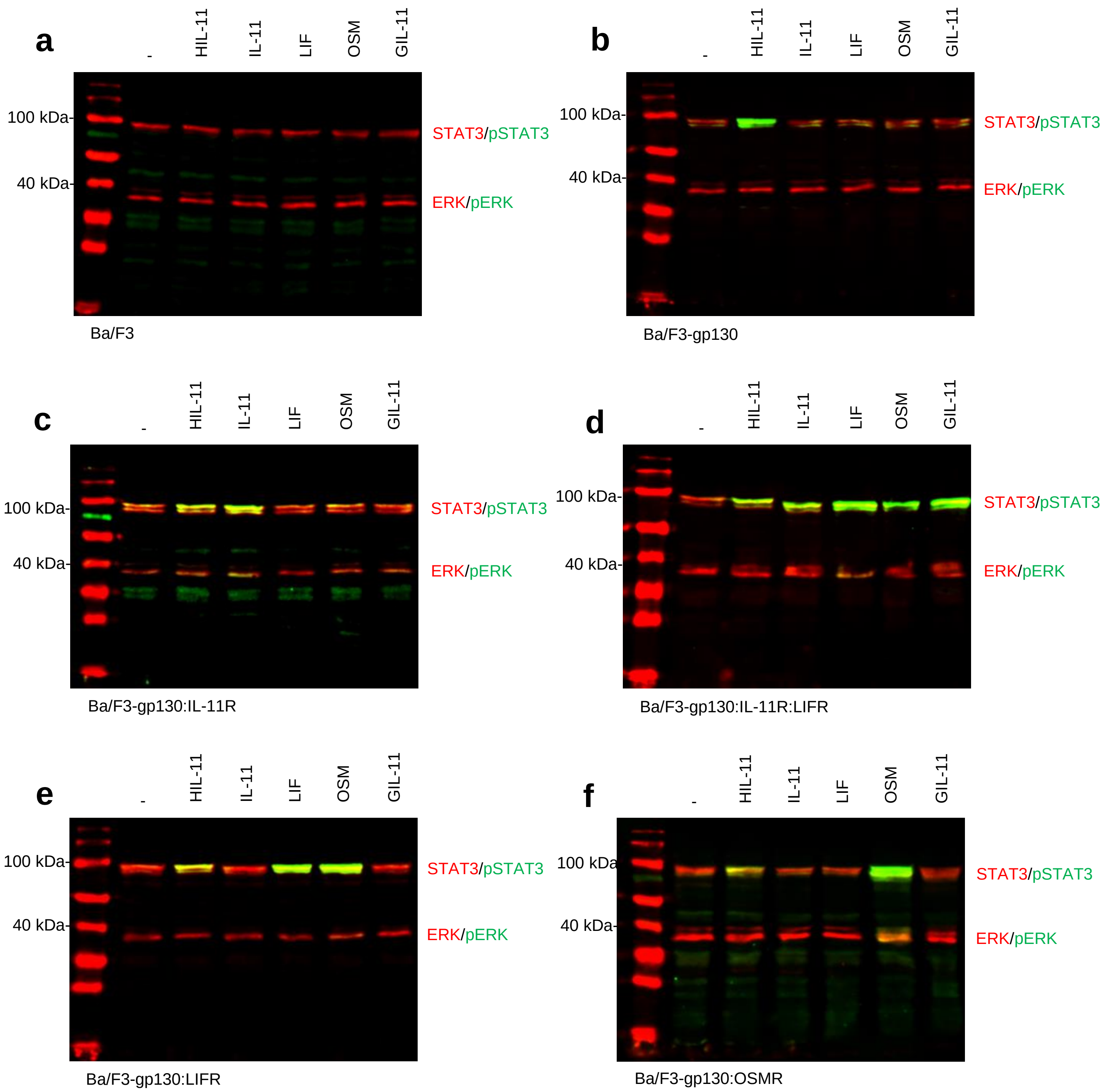

Supplemental Figure 2. Western Blots from figure 2b. STAT3 activation of **(a)** Ba/F3 **(b)** Ba/F3-gp130 **(c)** Ba/F3-gp130:IL11R **(d)** Ba/F3-gp130:IL11R:LIFR **(e)** Ba/F3-gp130:LIFR and **(f)** Ba/F3-gp130:OSMR cells without cytokine (-) and after stimulation with 50 ng/ml HIL-11, 50 ng/ml IL-11, 10 ng/ml LIF, 10 ng/ml OSM, 500 ng/ml GIL-11 for 15 min. Equal amounts of proteins (50 µg/lane) were analyzed via specific antibodies detecting phospho-STAT3 and STAT3. Western blot data shows one representative experiment out of three.

Supplementary Figure 3

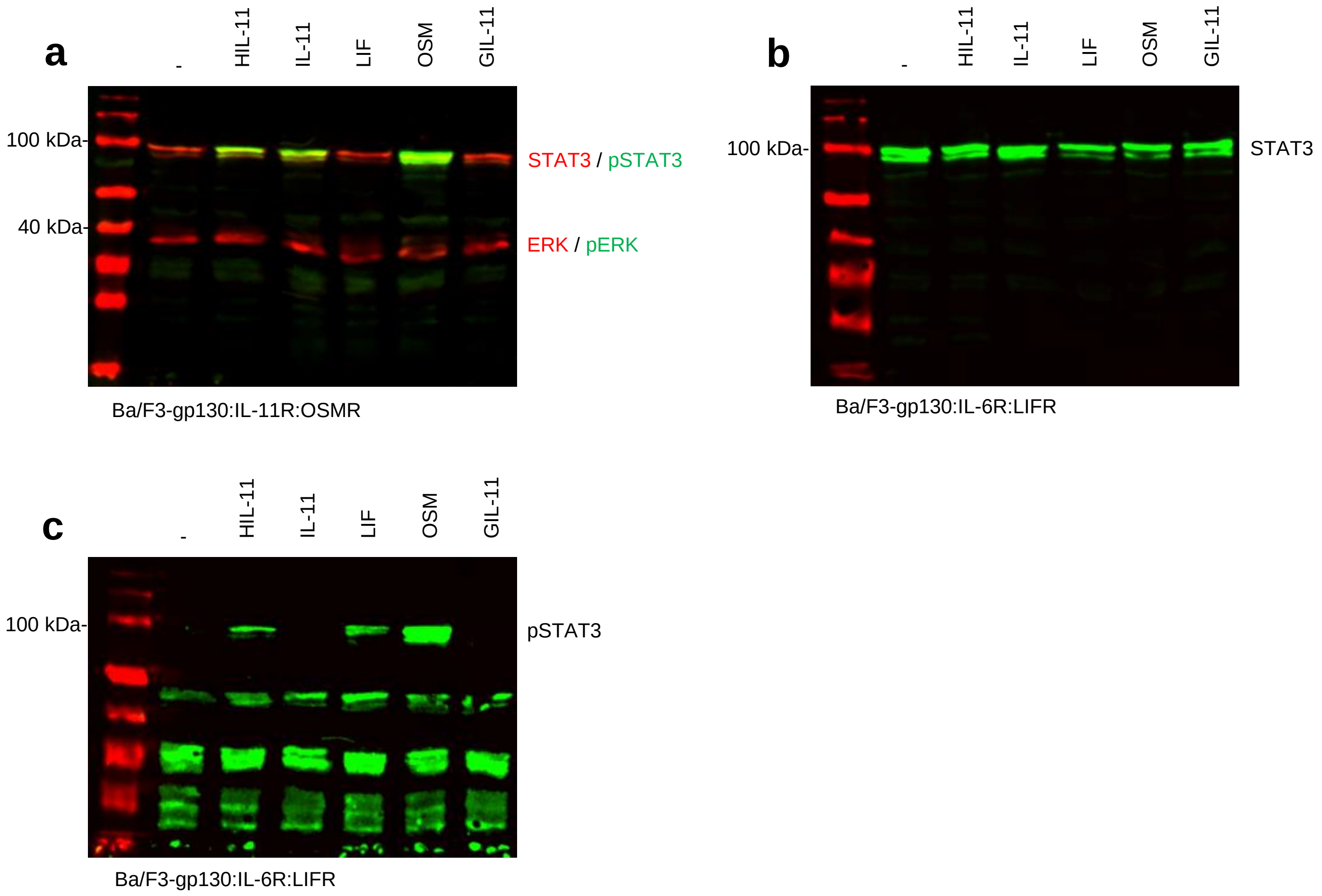

Supplemental Figure 3. Western Blots from figure 2b. STAT3 activation of **(a)** Ba/F3-gp130:IL11R:OSMR **(b)** Ba/F3-gp130:IL6R:LIFR **(c)** Ba/F3-gp130:IL-6R:LIFR cells without cytokine (-) and after stimulation with 50 ng/ml HIL-11, 50 ng/ml IL-11, 10 ng/ml LIF, 10 ng/ml OSM, 500 ng/ml GIL-11 for 15 min. Equal amounts of proteins (50 µg/lane) were analyzed via specific antibodies detecting phospho-STAT3, STAT3, ERK and phospho-ERK. Western blot data shows one representative experiment out of three.

Supplementary Figure 4

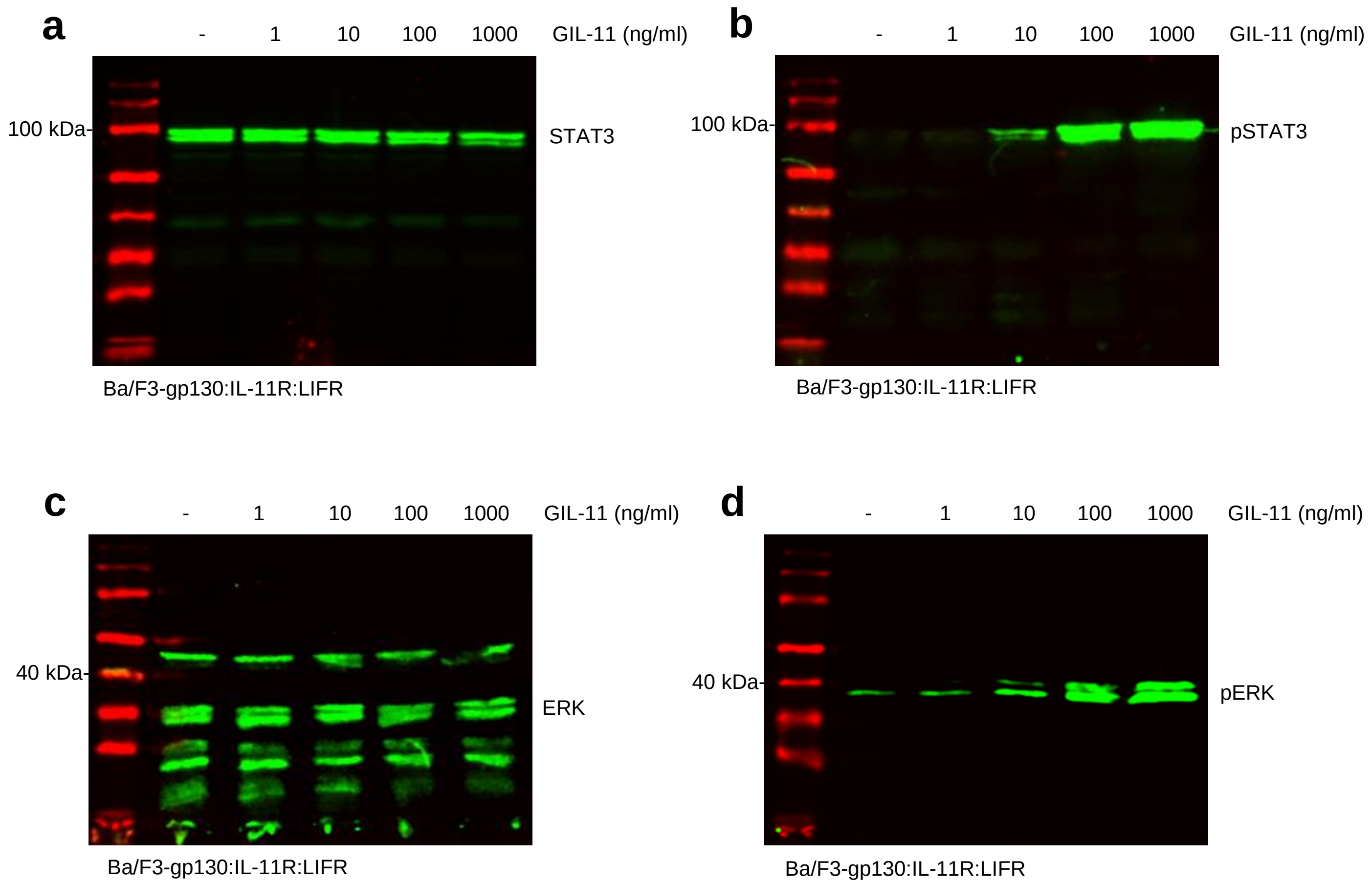

Supplemental Figure 4. Western Blots from figure 4d. Wetern Blot analysis of **(a)** STAT3 **(b)** phospho-STAT3 **(c)** ERK and **(d)** phospho-ERK in Ba/F3-gp130:IL11R:LIFR cells with increasing concentrations of GIL-11 for 15 min. Equal amounts of proteins (50 µg/lane) were analyzed via specific antibodies detecting phospho-STAT3, STAT3, ERK and phospho-ERK. Western blot data shows one representative experiment out of three.

Supplementary Figure 5

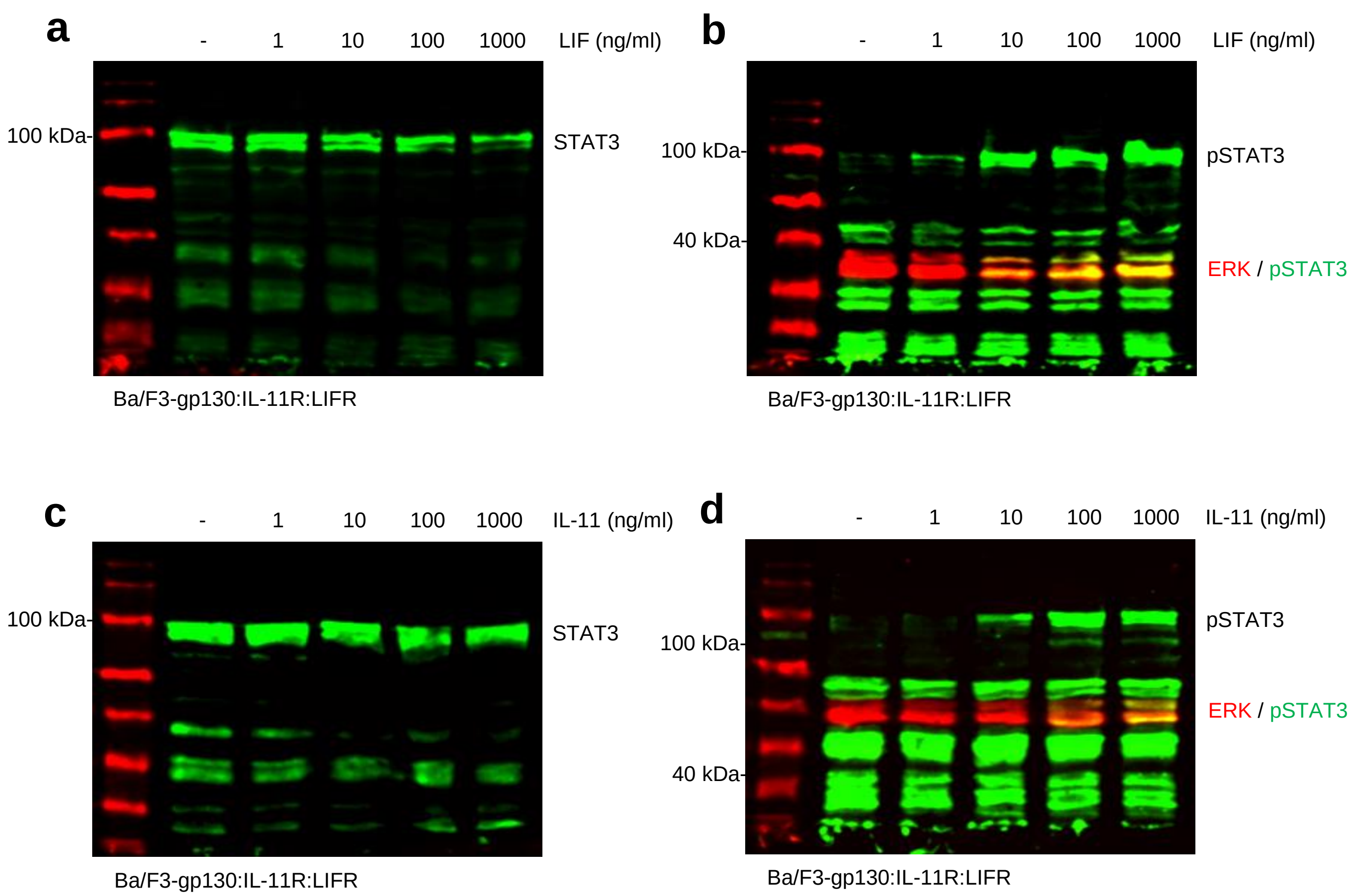

Supplemental Figure 5. Western Blots from figure 4d. Wetern Blot analysis of **(a)** STAT3 and **(b)** phospho-STAT3, ERK and phosphoERK in Ba/F3-gp130:IL11R:LIFR cells with increasing concentrations of LIF. Wetern Blot analysis of **(c)** STAT3 and **(d)** phospho-STAT3, ERK and phosphoERK in Ba/F3-gp130:IL11R:LIFR cells with increasing concentrations of IL-11 for 15 min. Equal amounts of proteins (50 µg/lane) were analyzed via specific antibodies detecting phospho-STAT3, STAT3, ERK and phospho-ERK. Western blot data shows one representative experiment out of three.

Supplementary Figure 6

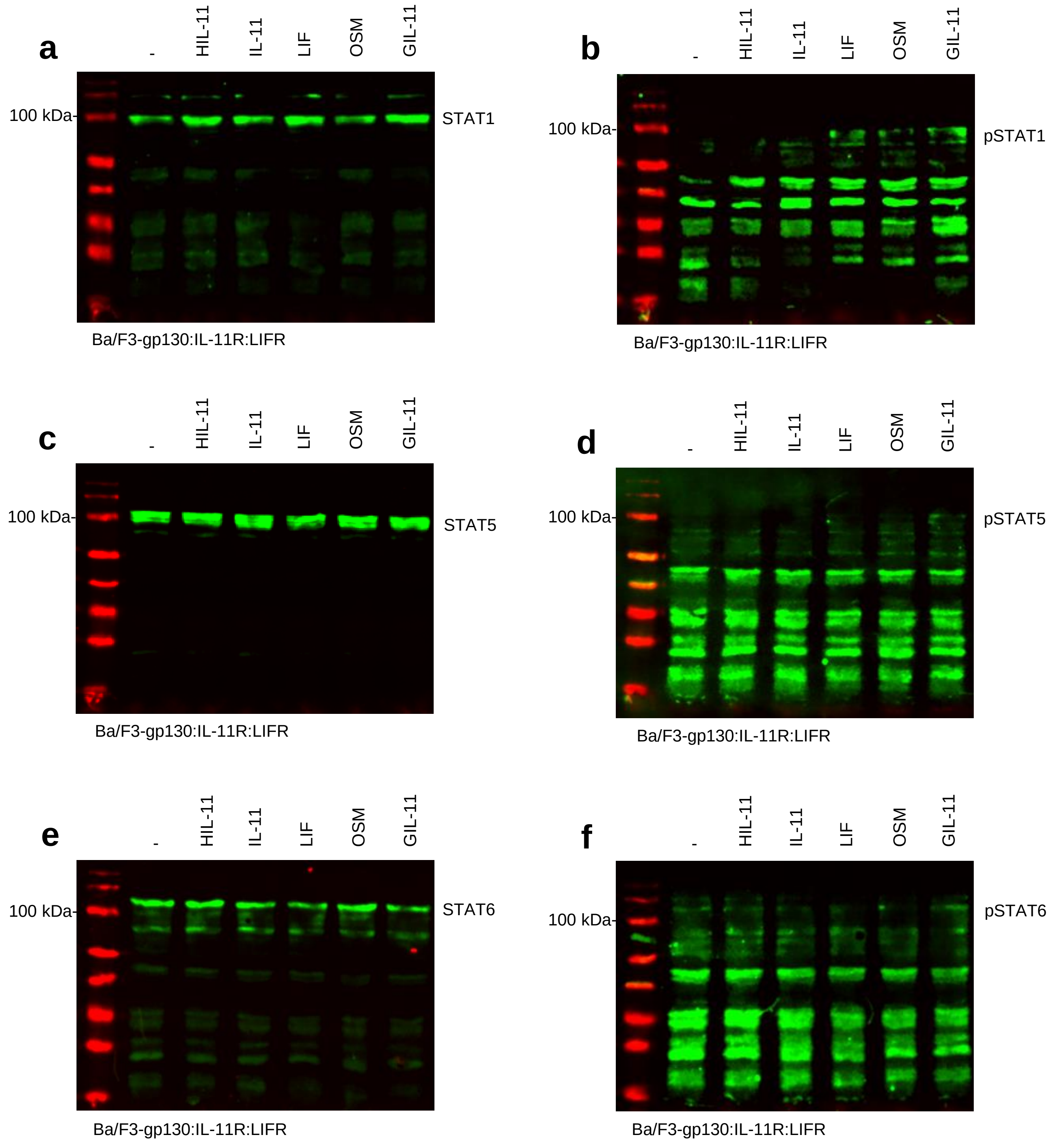

Supplemental Figure 6. Western Blots from figure 4e. Wetern Blot analysis of **(a)** STAT1 **(b)** phospho-STAT1 **(c)** STAT5 **(d)** phospho-STAT5 **(e)** STAT6 and **(f)** phospho-STAT6 in Ba/F3-gp130:IL11R:LIFR cells without cytokine (-) and after stimulation with 50 ng/ml HIL-11, 50 ng/ml IL-11, 10 ng/ml LIF, 10 ng/ml OSM, 500 ng/ml GIL-11 for 15 min. Equal amounts of proteins (50 µg/lane) were analyzed via specific antibodies detecting phospho-STAT3 and STAT3. Western blot data shows one representative experiment out of three.

Supplementary Figure 7 According Figure 4 F

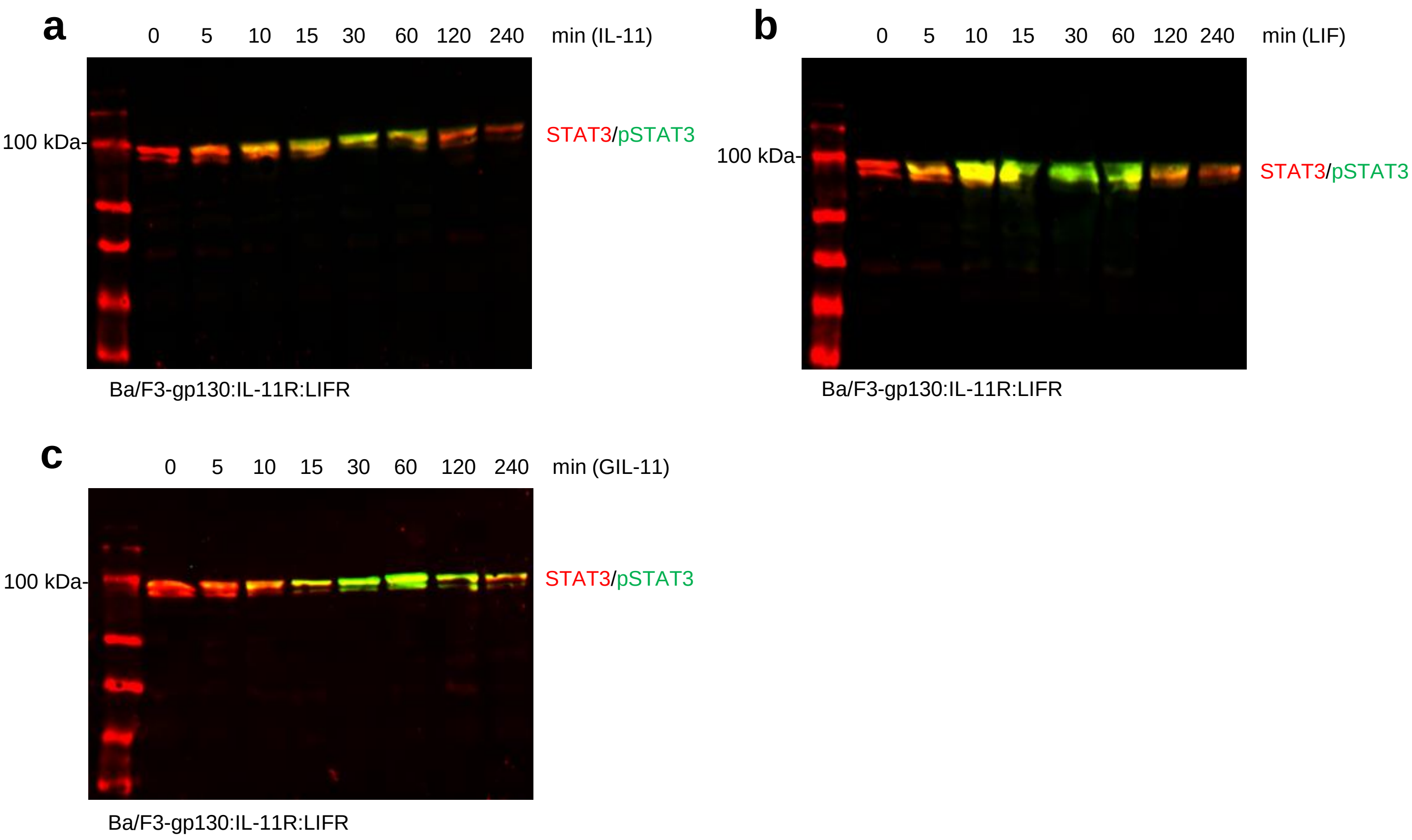

Supplemental Figure 7. Western Blots from figure 4f. Time-dependent STAT3 activation of Ba/F3-gp130:IL-11R:LIFR cells with **(a)** IL-11 (200 ng/ml) **(b)** LIF (20 ng/ml) and **(c)** GIL-11 (200 ng/ml) for the indicated time. Equal amounts of proteins (50 µg/lane) were analyzed via specific antibodies detecting phospho-STAT3 and STAT3. Western blot data shows one representative experiment out of three.

Supplementary Figure 8

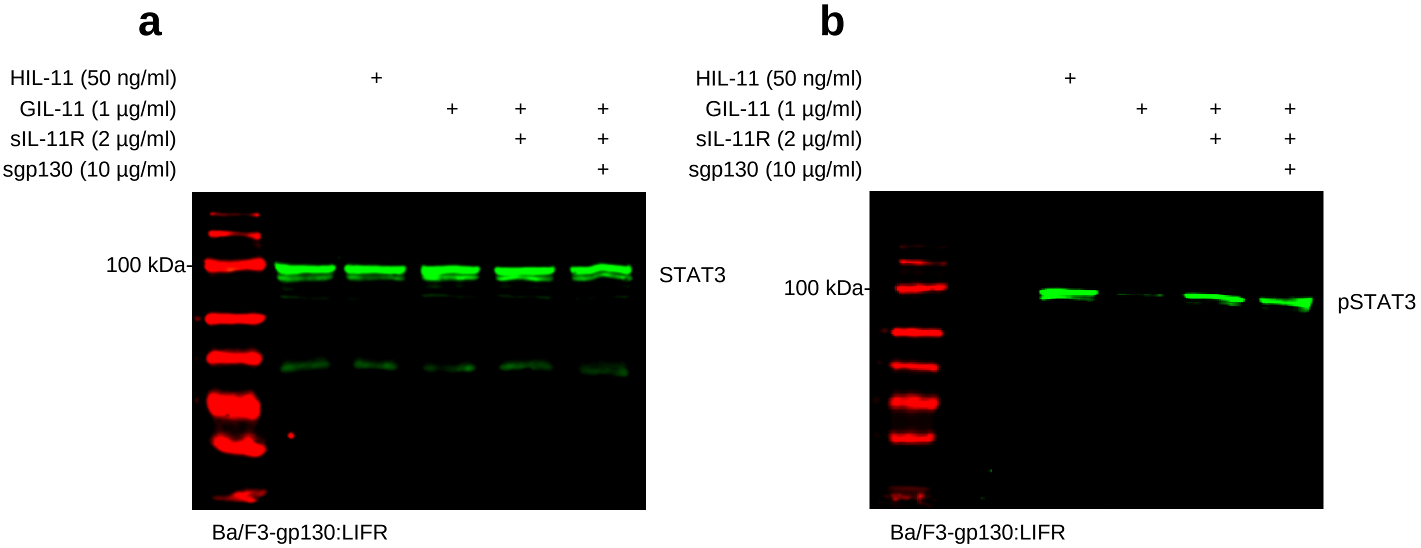

Supplemental Figure 8. Western Blots from figure 5c. Wetern Blot analysis of **(a)** STAT3 and **(b)** phospho-STAT3 in Ba/F3-gp130:LIFR cells without cytokine (-) and after stimulation with HIL-11 (50 ng/ml), GIL-11 (1 µg/ml), GIL-11 (1 µg/ml):sIL-11R (2 µg/ml), GIL-11 (1 µg/ml):sIL-11R (2 µg/ml):sgp130Fc (10 µg/ml) for 15 min. Equal amounts of proteins (50 µg/lane) were analyzed via specific antibodies detecting phospho-STAT3 and STAT3. Western blot data shows one representative experiment out of three.

Supplementary Figure 9

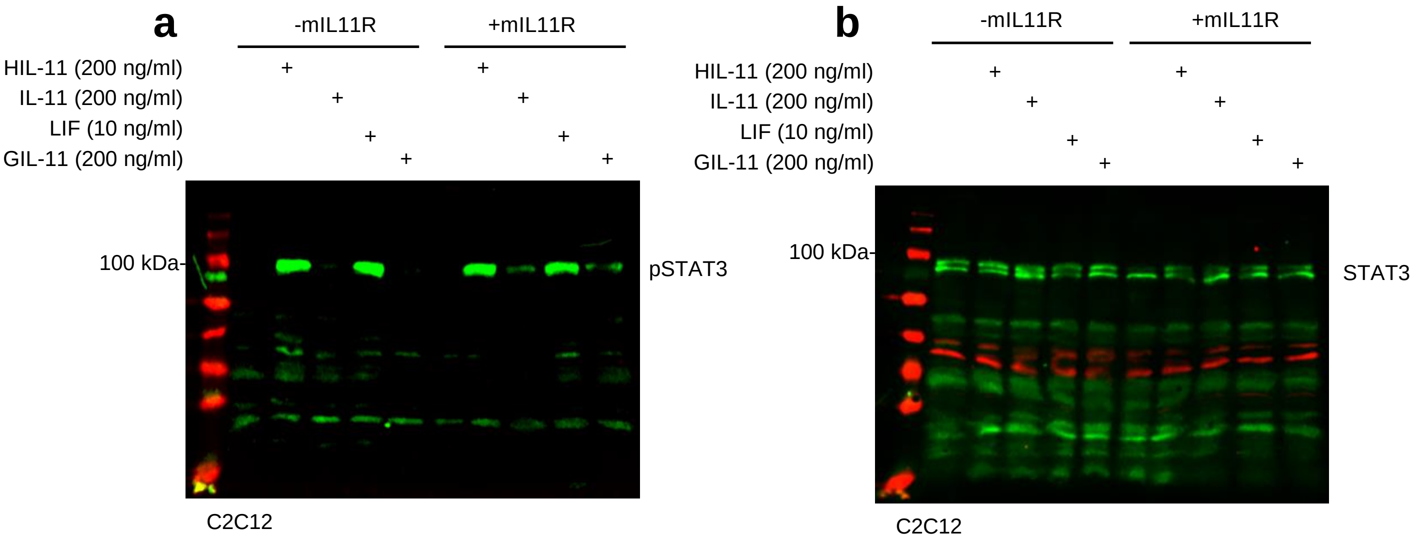

Supplemental Figure 9. Western Blots from figure 6a. Wetern Blot analysis of **(a)** phospho-STAT3 and **(b)** STAT3 in un-transfected and transfected with a cDNA coding for murine IL-11R murine myoblasts (C2C12) without cytokine (-) and after stimulation with HIL-11 (200 ng/ml), IL-11 (200 ng/ml), LIF (10 ng/ml), GIL-11 (200 ng/ml) for 15 min. Equal amounts of proteins (50 µg/lane) were analyzed via specific antibodies detecting phospho-STAT3 and STAT3. Western blot data shows one representative experiment out of three.

Supplementary Figure 10

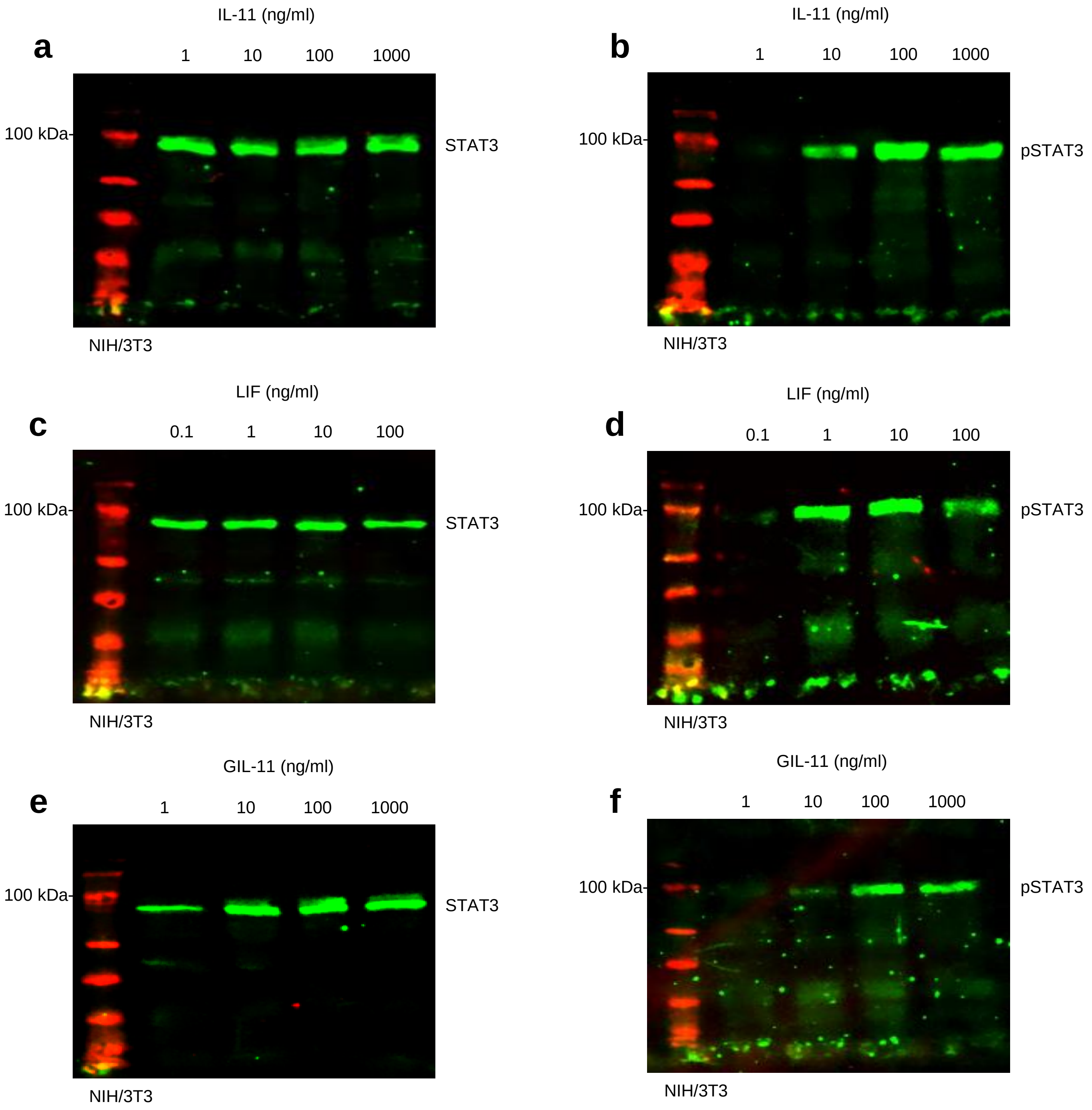

Supplemental Figure 10. Western Blots from figure 6b. Wetern Blot analysis of **(a)** STAT3 and **(b)** phospho-STAT3 in murine fibroblast NIH/3T3 after stimulation with increasing amounts of IL-11 (1, 10, 100, 1000 ng/ml). **(c)** STAT3 and **(d)** phospho-STAT3 in murine fibroblast NIH/3T3 after stimulation with increasing amounts of LIF (0.1, 1, 10, 100 ng/ml). **(e)** STAT3 and **(f)** phospho-STAT3 in murine fibroblast NIH/3T3 after stimulation with increasing amounts of GIL-11 (1, 10, 100, 1000 ng/ml) for 20 min. Equal amounts of proteins (50 µg/lane) were analyzed via specific antibodies detecting phospho-STAT3 and STAT3. Western blot data shows one representative experiment out of three.

Supplementary Figure 11

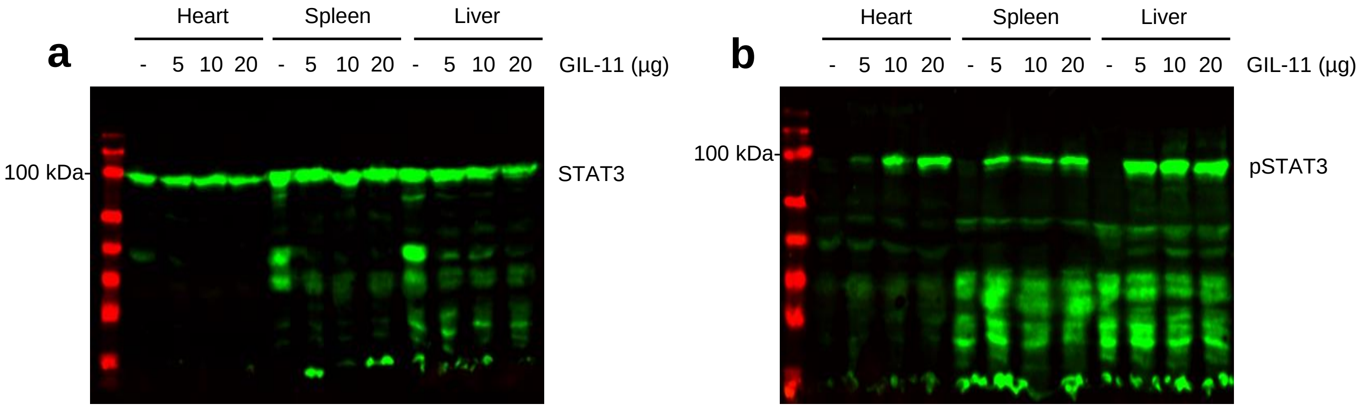

Supplemental Figure 11. Western Blots from figure 6c. Wetern Blot analysis of **(a)** STAT3 and **(b)** phospho-STAT3 in heart, liver and spleen after injection of 5, 10 or 20 µg/ml GIL-11 of wild-type mice. Mice were sacrificed 30 min after intraperitoneal cytokine injection. Equal amounts of proteins (50 µg/lane) were analyzed via specific antibodies detecting phospho-STAT3 and STAT3. Western blot data shows one representative experiment out of three.
