# Supplementary material for: Cytokimera GIL-11 rescued IL-6R deficient mice from partial hepatectomy-induced death by signaling via non-natural gp130:LIFR:IL-11R complexes
Source: Commun Biol. 2023 Apr 15;6:418. doi: 10.1038/s42003-023-04768-4 (PMC10105715; doi:10.1038/s42003-023-04768-4)
Supplement: Supplementary file 2 — Description of Additional Supplementary Files [file 42003_2023_4768_MOESM2_ESM.pdf]

Description of Additional Supplementary Files

File name: Supplemental Data
Description: Supplementary raw data belonging to the graphs.
